# Identification of Genomic Associations for Adult Plant Resistance in the Background of Popular South Asian Wheat Cultivar, PBW343

**DOI:** 10.3389/fpls.2016.01674

**Published:** 2016-11-08

**Authors:** Huihui Li, Sukhwinder Singh, Sridhar Bhavani, Ravi P. Singh, Deepmala Sehgal, Bhoja R. Basnet, Prashant Vikram, Juan Burgueno-Ferreira, Julio Huerta-Espino

**Affiliations:** ^1^International Maize and Wheat Improvement Center (CIMMYT)Texcoco, Mexico; ^2^Institute of Crop Science, Chinese Academy of Agricultural SciencesBeijing, China; ^3^Campo Experimental Valle de México, Instituto Nacional de Investigaciones Forestales, Agrícolas y Pecuarias, Universidad Autónoma ChapingoTexcoco, Mexico

**Keywords:** wheat, rust resistance, nested association mapping (NAM), genetic similarity, joint linkage analysis, quantitative traits loci (QTL)

## Abstract

Rusts, a fungal disease as old as its host plant wheat, has caused havoc for over 8000 years. As the rust pathogens can evolve into new virulent races which quickly defeat the resistance that primarily rely on race specificity, adult plant resistance (APR) has often been found to be race non-specific and hence is considered to be a more reliable and durable strategy to combat this malady. Over decades sets of donor lines have been identified at International Maize and Wheat Improvement Center (CIMMYT) representing a wide range of APR sources in wheat. In this study, using nine donors and a common parent “PBW343,” a popular Green Revolution variety at CIMMYT, the nested association mapping (NAM) population of 1122 lines was constructed to understand the APR genetics underlying these founder lines. Thirty-four QTL were associated with APR to rusts, and 20 of 34 QTL had pleiotropic effects on SR, YR and LR resistance. Three chromosomal regions, associated with known APR genes (*Sr58/Yr29/Lr46, Sr2/Yr30/Lr27*, and *Sr57/Yr18/Lr34*), were also identified, and 13 previously reported QTL regions were validated. Of the 18 QTL first detected in this study, 7 were pleiotropic QTL, distributing on chromosomes 3A, 3B, 6B, 3D, and 6D. The present investigation revealed the genetic relationship of historical APR donor lines, the novel knowledge on APR, as well as the new analytical methodologies to facilitate the applications of NAM design in crop genetics. Results shown in this study will aid the parental selection for hybridization in wheat breeding, and envision the future rust management breeding for addressing potential threat to wheat production and food security.

## Introduction

The global wheat (*Triticum aestivum* L.) demand is expected to increase by 60–110% to feed the population in 2050 (Tilman et al., 2011). Higher yield gains are required to meet the projected demand posed by increasing population and against the increasing production challenges from a host of biotic and abiotic stresses (Rajaram and Braun, 2008). Globally, the three wheat rusts, stem rust (SR), yellow rust (YR), and leaf rust (LR) are the most economically damaging diseases of the crop, inflicting losses of 60% or more, and are the constant threats to food security (Rajaram and Braun, 2008). This is due to their wide distribution, capacity to form new virulent races, ability to move long distances, and potential to develop rapidly under optimal environmental conditions. The UN Food and Agriculture Organization (FAO) estimates that 31 countries in East and North Africa, the Near East, Central and South Asia, accounting for more than 37% of global wheat production area, are at risk of wheat rust diseases. Furthermore, as wheat growing mega-environments shift with changing climate, there is risk of more severe rust infection in varieties suffering environmental stress emanating from hostile soil, pests and pathogens from remnant vegetation and other constraints.

The wheat SR, caused by fungus *Puccinia graminis* f. sp. *tritici* (*Pgt*), has historically been a menace to wheat production worldwide (Khan et al., 2013). A considerably newer *Pgt* race, TTKSK detected in Uganda in 1999 and commonly referred as Ug99, overcame the widely deployed resistance genes of wheat origin (Pretorius et al., 2000). Over the last decades, several variants of Ug99 were detected in Kenya (Jin et al., 2009), South Africa (Pretorius et al., 2010), and many other wheat growing countries of North- and South-Eastern African countries (Singh et al., 2015). The original race spread out into Yemen and Sudan in 2006, in Iran in 2007 and in Egypt in 2014 (Nazari et al., 2009; Singh et al., 2015). This has raised concern of a major epidemic that could cause damage in wheat growing countries on all continents as most popular varieties grown currently are susceptible to Ug99 race group. The wheat YR, caused by *P*. *striiformis* f. sp. *tritici* (*Pst*), affects up to 40% of the wheat production in countries such as Mexico, India, Pakistan, Bangladeshi, and China (Khan et al., 2013). Recent investigation by Beddow et al. (2015) has indicated that YR is one of the deadliest threats to global wheat production as the pathogen continues to rapidly evolve and spread across globe making nearly 88% of world's wheat susceptible and causing an estimated loss of 5.47 million tons of wheat grains annually. Recent YR epidemics across different continents have been mainly observed due to rapid adaptation of pathogen to newer geographical regions and relatively higher temperature, and due to rapid breakdown of widely deployed major genes (ICARDA, 2011; Basnet et al., 2014). The wheat LR, caused by *Puccinia triticina* (*Pt*), is also one of the most widely distributed diseases of wheat in the world, and can cause yield losses of up to 40% in susceptible cultivars by decreasing kernel number per spike and kernel weight (Khan et al., 2013).

In general, the rust resistance can be classified into two major types i.e., race-specific and race non-specific. Race specific resistance is often conferred by a single major gene which is inherited in simple Mendelian fashion. Such resistance is often detected at early seedling stage of plant growth and remains effective throughout whole life cycle, and hence it is also called “seedling or all-stage resistance.” In contrast, race non-specific resistance is conferred by multiple additive genes possessing quantitative inheritance and is expressed during post-seedling stage of plant growth. So, the race non-specific resistance is synonymously called as “Adult plant resistance (APR)” or “slow rusting resistance.” As APR is generally conferred by multiple additive genes, it is not subjected to regular “boom and bust cycle” of disease epidemics. Sources of quantitative resistance in crop plants, readily detected in post-seedling growth stages and associated with race non-specific resistance, have proven to be durable, making APR an important breeding target for long-term rust resistance (Knott, 1982; Parlevliet, 2002). Therefore, it is critical to deploy APR genes to rust diseases in high yielding varieties. The Global Wheat Program at International Maize and Wheat Improvement Center (CIMMYT), initiated to identify APR genes for wheat rust in early 1980's. But due to their small effects, it is difficult to follow them in breeding programs. Over decades prominent sets of donor lines have been identified as important sources of APR to wheat rusts which were more rigorously utilized after the inception of Durable Rust Resistance Wheat (DRRW) Project in 2005 under the umbrella of Borlaug Global Rust Initiative (BGRI). Series of bi-parental populations were developed by crossing these APR donor lines with the most popular Green Revolution variety, PBW343. These populations provided a solid foundation for APR resources to rusts resistance (including Ug99) wheat breeding program of CIMMYT. Although numerous rust resistant elite germplasm have been developed using these crosses, clear understanding of complex genetics underlying these APR donors still remains elusive.

Till now, almost all the genetic studies on rust resistance has relied on linkage analysis using bi-parental populations and association mapping in hundreds of wheat breeding lines (Rosewarne et al., 2013; Li et al., 2014; Yu et al., 2014). Several resistance genes have been identified and few of them (such as *Sr2, Lr34, Lr46*, and *Lr67*) are well characterized and widely used in breeding (Rosewarne et al., 2013; Li et al., 2014; Yu et al., 2014; Moore et al., 2015). Nested association mapping (NAM) design pioneered in maize (Buckler et al., 2009) combines the advantages of linkage analysis and association mapping through the development of a large number of recombinant inbred lines (RILs) from diverse founders for identifying QTL. It has been successfully used to dissect the genetic architecture of complex traits in maize including flowering time (Buckler et al., 2009), leaf traits (Tian et al., 2011), male and female inflorescence (Brown et al., 2011), and various disease resistance and quality traits (Poland et al., 2011; Cook et al., 2012). Thus, NAM is a powerful design to study the genetic architecture of complex traits. More recently, Bajgain et al. (2016) used a spring wheat NAM population, composed of 852 lines, to conduct a join linkage analysis for SR resistance QTL.

One of the goals of the wheat breeding program at CIMMYT is to develop new high yielding germplasm with durable resistance to rusts. Identification and transfer of new sources of race-specific resistance from various wheat relatives is also underway to enhance the diversity for resistance. Several sources of APR to Ug99 were identified in CIMMYT spring bread wheat germplasm and mapping studies have identified genomic regions that contribute to APR (Yu et al., 2011; Singh et al., 2013). Developing and use of molecular markers for APR can speed up selection processes and also provide opportunities to focus on other important traits simultaneously. The objectives of our study were: (1) to evaluate the genetic relatedness and phenotypic diversity of APR donor lines; (2) to map QTL associated with APR to SR, YR, and LR in the CIMMYT NAM population; (3) to identify the new resistance loci that could be useful in diversifying the current set of resistance genes by *In silico* analysis of QTL flanked marker sequences; and (4) to investigate the new analytical methodology for facilitating the applications of NAM design in crop genetics.

## Materials and methods

### CIMMYT wheat NAM population

**Figure 1:**
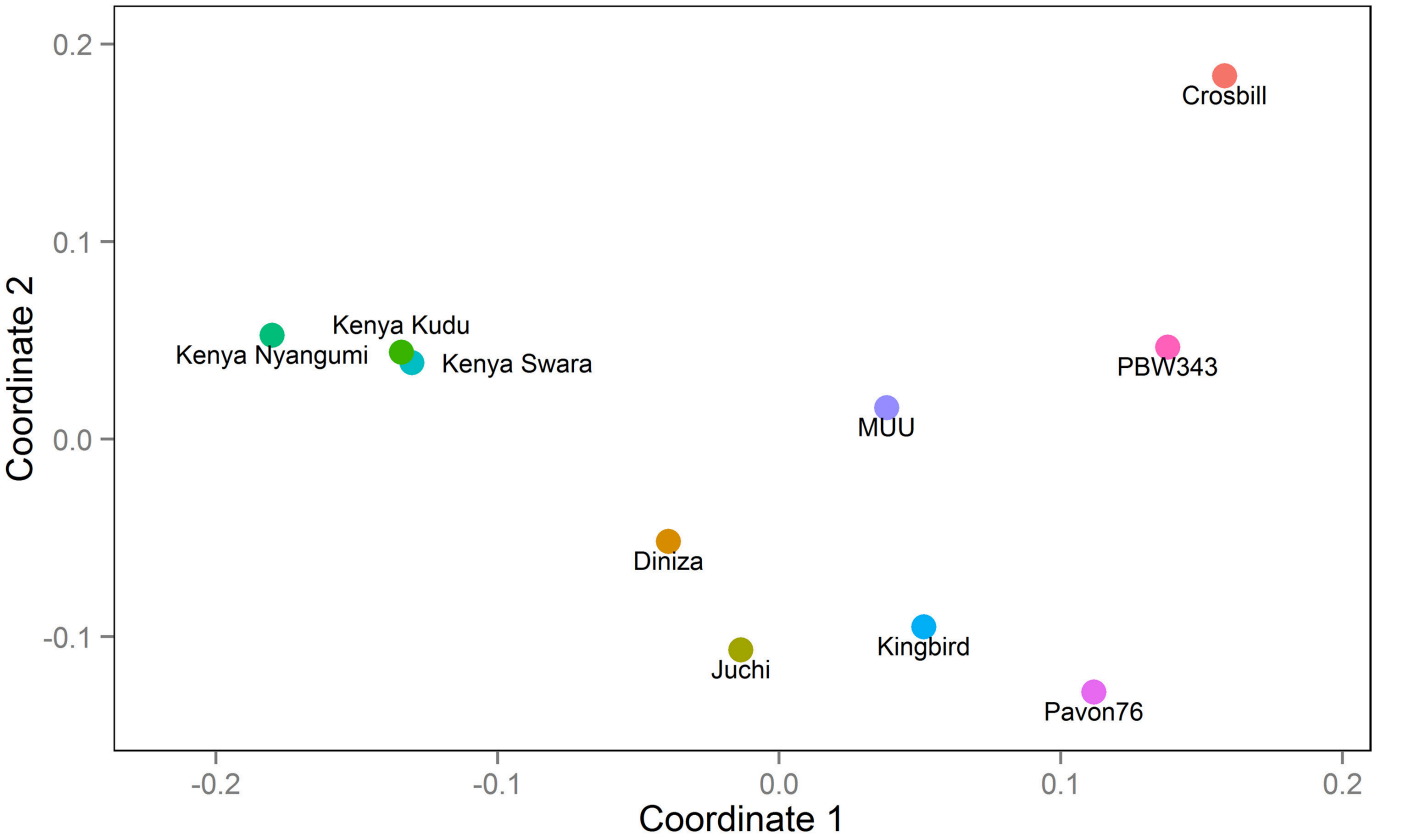
**Genetic relatedness among 10 founders: PBW343, Diniza, Crosbill, Juchi, Kenya Swara, Kingbird, Kenya Kudu, Pavon76, MUU, and Kenya Nyangumi**.

The CIMMYT wheat NAM population was composed of 1122 RILs derived from the crosses of a common parent (PBW343) with each of nine diverse founders. The nine founder lines were Diniza, Crosbill, Juchi, Kenya Swara, Kingbird, Kenya Kudu, Pavon76, Muu, and Kenya Nyangumi (Figure 1). The common parent, PBW343, was crossed to the other nine founders, and F_1_ plants were selfed to generate nine segregating F_2_ populations. Out of each F_2_ population, 80, 87, 90, 177, 88, 89, 178, 146, and 187 RILs were derived through single-seed descent with repeated selfing to the F_5_ of F_6_ generation for the nine families, respectively (Supplementary Table 1). To facilitate the illustration throughout the paper, the names of nine individual families are abbreviated as PB/DZ, PB/CB, PB/JC, PB/KS, PB/KB, PB/KK, PB/P76, PB/MU, PB/KN, respectively, and the nine founder lines, other than PBW343, are mentioned as “non-PBW343” in general. Moderately susceptible bread wheat (*Triticum aestivum*) key parent PBW343, is a selection (GID2430154) from CIMMYT line Attila with the pedigree Nord Deprez/VG9144//Kalyansona/Bluebird/3/Yaco/4/Veery#5 (Table 1). The nine non-PBW343 wheat lines carried high levels of APR to SR (Table 2) despite being susceptible to Ug99 race group in seedling growth stage.

**Table 1:** **Detailed information of released year and country, and pedigree of the ten founder lines**.

**Founder line**	**Released country, year**	**Pedigree**
PBW343	India, 1995	Nord Desprez/VG9144//Kalyansona/Bluebird/3/Yaco/4/Veery#5
Diniza	Mexico, 1999	Huac/Ti-R/3/Atr^*^2/7C//Nac/4/Sara/5/2^*^Parula/Vee#6//Myna/Vul
Crosbill	Mexico, 1999	Cndo/R143//Ente/Mexi_2/3/Aegilops squarrosa (taus)/4/Weaver/5/2^*^Kauz/6/Fret2
Juchi	Mexico, 1999	Kite/Bobwhite/3/Mon//Sis/Can
Kenya Swara	Kenya, 1972	PI59284/3/PP-Aus//Ifife/Etawah^*^2/4/Swd/T.timopheevii//K^*^2/3/Y59.2.B
Kingbird	Mexico, 1999	TAM200/Tui/6/Pavon 76//CAR422/Ana/5/Bobwhite/Crow//Buc/Pavon 76/3/Yr/4/Trap#1
Kenya Kudu	Kenya, 1966	Fife/2^*^White Naples//Ifife/Eden/3/A8/4/Kr/Mq//Kenya 73D
Pavon76	Mexico, 1976	Vcm//CNO67/7C/3/Kal/Bb
Muu	Mexico, 1999	Pfau/Weaver^*^2/11/Weaver/9/Kt/Bage// Fn/U/3/Bza/4/Trm/5/Aldan/6/Seri/7/Vee#10/8/Opata/10/Borlaug95
Kenya Nyangumi	Kenya, 1979	Tzpp//Ske/LR64A/3/Afm/4/Kenya Swara/K4500

**Table 2:** **Parents' performance, means, ranges, and the heritability in the broad sense (***H***^**2**^) of stem rust severity in nine families of the CIMMYT NAM**.

**Family**	**Parent mean**		**Progeny**					***H*^2^**
	**PBW343**	**Non-PBW343**	**No. RILs**	**No. trials**	**Mean**	**Std**.	**Range**	
PB/DZ	63.2	15.0	80	2	35.6	19.0	5–85	0.55
PB/CB	63.2	12.1	87	4	24.1	19.1	1–75	0.57
PB/JC	63.2	22.5	90	2	36.1	17.7	2–90	0.45
PB/KS	63.2	10.0	177	2	27.2	23.2	0–100	0.78
PB/KB	63.2	7.0	88	3	31.7	19.3	1–80	0.68
PB/KK	63.2	10.0	89	3	37.4	19.9	1–85	0.51
PB/P76	63.2	6.6	178	3	26.9	17.8	1–80	0.54
PB/MU	63.2	5.0	146	4	28.4	19.5	0–100	0.53
PB/KN	63.2	5.0	187	2	29.6	23.4	0–90	0.62

*Std. is the standard deviation of the phenotype for each family*.

### Evaluating SR, YR, and LR severity

The 10 founder parents, highly susceptible bread wheat check variety “Cacuke” and the CIMMYT wheat NAM population were evaluated for SR severities at the Kenya Agricultural Research Institute (KARI) in Njoro during four crop seasons: main season 2009, main and off-seasons 2010, and main season 2011, hereafter denoted as SR-MS2009, SR-MS2010, SR-OS2010, and SR-MS2011, respectively (Supplementary Table 1). The RILs and parents were sown using a randomized complete block design with two replicates. Field plots consisted of two 1-m rows spaced 20 cm apart with a 0.5-m pathway. Approximately 60–70 seeds were sown in each plot. The experimental block was surrounded by a spreader row consisting of varieties differentially susceptible to the *Sr24* virulent variant race TTKST. Hill plots of spreaders were also planted in the middle of the pathway on one side of each plot to facilitate uniform disease build-up and spread. On at least two occasions just prior to booting, freshly collected urediniospores suspended in distilled water were injected into culms in the spreader plots (1–3 plants/m) using a hypodermic syringe. Disease response in the field was assessed twice. First when the susceptible check variety Cacuke displayed 50–60% SR severity and subsequently at peak disease development, when Cacuke displayed 100% SR at the mid-dough stage of plant growth. Percent disease severity was scored using the modified Cobb Scale (Peterson et al., 1948). The second rating was considered as the phenotype in this study. All the nine families were evaluated for APR to SR during two seasons of 2010 (Supplementary Table 1). Five of them (i.e., PB/CB, PB/KB, PB/KK, PB/P76, and PB/MU) were screened for APR to SR at SR-MS2009, while two of them (i.e., PB/CB and PB/MU) were screened for APR to SR at MS-2011.

Parents and population lines were evaluated for YR under field conditions in rust nurseries operated by CIMMYT near Toluca, Edo. Mexico, Mexico, and in Njoro, Kenya, in 2010 and 2011, which are denoted as YR-T2010, YR-T2011, YR-K2010, and YR-K2011, respectively (Supplementary Table 1). Two replicates of parents and RILs were assessed in each trial. YR severity in each plot was visually scored (anthesis - milk stage) using the modified Cobb Scale (Peterson et al., 1948). All the nine families were evaluated for APR to YR at YR-T2010 (Supplementary Table 1). Five of them (i.e., PB/DZ, PB/CB, PB/JC, PB/KB, and PB/MU) were screened for APR to YR at YR-K2010, while only one of them was screened for APR to YR at each of YR-T2011 (i.e., PB/KK), and YR-K2011 (i.e., PB/MU).

For LR screening, parents and RILs were evaluated in field nurseries operated by CIMMYT in Ciudad Obregon, Sonora, Mexico, in 2010, 2011, and 2012, denoted as LR-2010, LR-2011, and LR-2012, respectively (Supplementary Table 1). Replicated trials with parents and RILs were grown in Obregon. Each plot was visually scored around early-dough stage for LR severity with the percentage of leaf covered with disease infection calculated as described for YR. Five of the nine families (i.e., PB/DZ, PB/CB, PB/JC, PB/P76, and PB/KN) were evaluated for APR to LR at LR-2010 (Supplementary Table 1), four of them (i.e., PB/CB, PB/KB, PB/KK, and PB/MU) were screened for APR to LR at LR-2011, and two of them (i.e., PB/KS and PB/KK) were screened for APR to LR at LR-2012. Phenotypic distributions of rust resistance of CIMMYT NAM population are shown in Supplementary Figure 1.

### Heritability in broad sense

An analysis of variance for phenotypic variance ($\sigma_{P}^{2}$) of the three rust resistances were estimated by mixed linear model using PROC MIXED of SAS software (Release 9.4; SAS Institute, Cary, NC, USA). Genotype, trials, and genotype by trial interactions were all considered as random effects, their variance were denoted as $\sigma_{G}^{2}$, $\sigma_{E}^{2}$, $\sigma_{GxE}^{2}$, respectively. It is generally agreed that environmental variance should not be included in the calculation of heritability (Holland et al., 2003). Phenotypic variance per plot in multi-trials can be written as $\sigma_{P}^{2}=\sigma_{G}^{2}+\sigma_{GxE}^{2}+\sigma_{\varepsilon }^{2}$, where $\sigma_{\varepsilon }^{2}$ is the variance of residual. Heritability in broad sense on an individual plot basis was thus calculated as (Holland et al., 2003), $H^{2}=\frac{\sigma_{G}^{2}}{\sigma_{G}^{2}\;+\;\sigma_{GxE}^{2}\;+\;\sigma_{\varepsilon }^{2}}$.

### Molecular analysis

DNA was extracted from lyophilized leaf tissue following the procedure described by Singh and Bowden (2011). A Nano-Drop ND8000 spectrophotometer (Thermo Fisher Scientific Inc, USA) was used for quantification of DNA samples. For Diversity Arrays Technology (DArT) genotyping, 500–1000 ng of restriction grade DNA, suspended in TE with a final concentration of 50–100 ng/μL were sent to Triticarte Pty. Ltd., Canberra, Australia (http://www.diversityarrays.com) for genome profiling (Neumann et al., 2011). Loci were scored as present (1) or absent (0). The overall call rate for the population was approximately 95% and the *Q*-values (estimates of marker quality) for most markers were above 80%.

### Linkage map and consensus map construction

For constructing a linkage map, there are two general steps, grouping and ordering. For marker grouping, the agglomerative hierarchical clustering algorithm (Day and Edelsbrunner, 1984) was used, with the significance of recombinant frequency between markers as the statistics to evaluate the relatedness among markers. After all markers were grouped, within each group nearest neighboring algorithm was used for map construction and two-opt was used for map improvement (Muyldermans et al., 2005). Finally, the linkage map was fine-tuned by permutation of a window of *m* markers (*m* = 5 in this study) and comparison of all *m*! possible maps. SARF (Sum of Adjacent Recombination Frequencies) (Falk and Chakravarti, 1992) was used as the rippling criteria.

For the consensus map construction in the CIMMYT NAM population, a similar strategy as used in the maize NAM and *Arabidopsis* NAM populations (McMullen et al., 2009; Li et al., 2011) was adopted by grouping and ordering algorithms described above. The PBW343 allele was designated as the “A” allele, the other nine non-PBW343 parent alleles were designated as the “B” allele, and the heterozygous loci were converted to missing data. Markers that were non-polymorphic in a particular family were converted to missing data. A total of 2193 genetic markers showed polymorphism between PBW343 and the other nine non-PBW343 parents (Supplementary Figure 2). 830 markers polymorphism in at least 3 families were used to construct the consensus map. 53 of 830 markers cannot be linked with the rest of markers, so were deleted from the dataset. Software JoinMap (Stam, 1993) and QTL IciMapping (Li et al., 2007) were used to validate the nine linkage maps and consensus map as well. Genotypic similarity was calculated by Flapjack (Milne et al., 2010; downloaded from https://ics.hutton.ac.uk/flapjack/). 272 SSR markers (Supplementary Table 2) were used to calculate the similarities among 10 founders. 777 DArT markers on the consensus map were used to calculate the similarities of CIMMYT NAM population.

### QTL mapping in single family

QTL were mapped in each of the single CIMMYT NAM family using inclusive composite interval mapping (ICIM), which was implemented in QTL IciMapping (Li et al., 2007). ICIM first determined a set of cofactors using stepwise regression to fit individual marker, and then scanned the entire genome at 1 cM intervals using maximum likelihood to test putative QTL at each point. In stepwise regression, the probability for marker effects entering into the model was set as 0.01, which was determined by 1000 times of permutation test and *quantile*-*quantile* (*QQ*) plot (Supplementary Figures 3, 4). The probability of a marker moving out of the model was set at twice the probability of a marker moving into the model. The LOD threshold to declare the existence of a QTL was calculated by 1000 times of permutation test using SR-MS2010 in nine RIL families. Permutation tests revealed LOD thresholds of 3.43, 3.46, 3.43, 5.19, 4.69, 4.31, 3.37, 3.14, and 3.35 for PB/DZ, PB/CB, PB/JC, PB/KS, PB/KB, PB/KK, PB/P76, PB/MU and PB/KN, respectively. Considering that thresholds retained from permutation tests are always conservative (Anderson and ter Braak, 2003), a LOD threshold of 2.5 was used to report QTL and determine common QTL across trials and populations. The phenotypic variance explained (PVE) by each QTL within each RIL family was calculated as described in Li et al. (2008).

### Joint QTL linkage mapping on CIMMYT NAM population

Joint inclusive composite interval mapping (JICIM; Li et al., 2011) was used to map QTL on CIMMYT NAM population, which was implemented in QTL IciMapping as well. The basic idea of JICIM was similar as that of ICIM, but in the first step a family main effect was fit first in the joint stepwise regression model followed by the selection of marker effects to enter or exit the model. In the joint stepwise regression, marker effects entered or exited the model based on the significance level chosen from running a permutation procedure 1000 times to control the Type I error rate at α = 0.05 (Anderson and ter Braak, 2003). The resulting 1000 *P*-values were sorted, and the 50th smallest *P*-value was selected as the empirical α = 0.05 entry threshold. Since for traits across trials the population size was different (Supplementary Table 1), permutation test was conducted per trait per trial. In this sense, the 50th smallest *P*-value was retained for 11 traits/trials, 8.9 × 10^−5^, 1.5 × 10^−4^, 8.6 × 10^−5^, 8.5 × 10^−5^, 6.9 × 10^−5^, 9.7 × 10^−5^, 9.7 × 10^−5^, 8.6 × 10^−5^, 8.6 × 10^−5^, 8.4 × 10^−5^, and 6.5 × 10^−5^ for SR-MS2009, SR-MS2010, SR-OS2010, SR-MS2011, YR-T2010, YR-K2010, YR-T2011, YR-K2011, LR-2010, LR-2011, and LR-2012 (Supplementary Table 1), respectively. Therefore, 1.0 × 10^−5^ was set as the probability for markers moving into the model. The probability of a marker moving out of the model was set at twice the probability of a marker moving into the model.

The LOD threshold to declare the existence of a QTL was calculated by permutation tests as well. Permutation tests revealed LOD thresholds of 4.50, 5.50, 3.50, 3.50, 3.50, 3.50, 3.50, 3.53, and 3.51 for SR-MS2009, SR-MS2010, SR-OS2010, SR-MS2011, YR-T2010, YR-K2010, YR-T2011, YR-K2011, LR-2010, LR-2011, and LR-2012 (Supplementary Table 1), respectively. An LOD threshold of 4.0 was used to report QTL and determine common QTL across trials and populations. QTL, having LOD score in the range of 3.0–4.0, and with pleiotropic effect with other QTL having LOD score higher than 4.0, were also reported. The PVE by each QTL in the NAM population was calculated as described in Li et al. (2011).

### Epistasis

For epistatic QTL mapping, we tested all possible pairs of scanning positions by ICIM (Li et al., 2008). That is to say, we can detect digenic interactions regardless of whether the two interacting QTL have significant additive effects or not. Due to the large amount of variables in digenic QTL mapping, we used a much stricter probability (1.0 × 10^−4^) of a marker moving into the model. The probability of a marker moving out of the model was set at twice the probability of a marker moving into the model. An empirical LOD threshold of 4.0 was used to declare the existence of epistatic QTL.

### Pleiotropy

A central issue in evaluating pleiotropy in linkage populations is determining whether correlated effects are the product of linked loci or the same gene. In this study, we determined pleiotropy by the co-localization of the QTL and the correlations of effects estimated at each locus to evidence that the same QTL were responsible. If two QTL were within 20 cM apart from each other, they were declared as the co-localized QTL. We correlated the effects at each locus against one another for each rust disease. Those with significantly correlated effects are likely to have the same genes and allele series that are producing the correlation. Counts of significant correlation were determined with *P* = 0.05, however, the significant loci were frequently much more significant.

### Prediction

We used the significant NAM QTL additive effect estimates to predict the rust resistance of the non-PBW343 founder lines (Buckler et al., 2009) by equation ${\hat{P}}_{j}=\mu +\sum_{i=1}^{q}a_{ij}$, where ${\hat{P}}_{j}$ is the predicted phenotype of the *j*th non-PBW343 founder in the *j*th family (*j* = 1, …, 9 in this study), μ is the population mean, *q* is the number of QTL, and *a*_*ij*_ is the additive effect estimate of *i*th QTL in *j*th family, and equals to 0 if the additive effect estimate was not significant in some families.

### *In silico* analysis

The sequences of the DArT markers were used as the query for BLAST in IWGSC portal (https://urgi.versailles.inra.fr/blast/blast.php) to retrieve the contigs. Top 5 hits with similarity percentage of the query were used as query in BLASTX searches in NCBI database querying wheat (*Triticum aestivum* L.), Brachypodium (*Brachypodium distachyon* (L.) *P. Beauv*), *Hordeum vulgare* L., and rice (*Oryza sativa* L.) databases. R genes encoding proteins that recognize pathogen effectors or their modified host targets were used to narrow down the results. For example, proteins characterized by the presence of motifs such as leucine-rich repeat (LRR), NBS-LRR (nucleotide binding site containing LRR), RLP (receptor like proteins coupled with extracellular LRR), resistance gene analogs (RGA) and RLK (receptor like kinase) were targeted.

## Results

### Phenotypic variability

Across the CIMMYT NAM population, the largest phenotypic variance was observed for SR, followed by YR, and LR (Tables 2–**4**; Supplementary Figure 1). The ten founder lines showed a wide range of phenotypic variation, especially for resistance to SR. Each family was evaluated for SR at least twice across different growing seasons in Kenya (Table 2). The common reference parent, PBW343, was moderately susceptible to SR compared with the other nine founders. The three families with the highest mean SR severity (%) were PB/KK, PB/JC, and PB/DZ. Transgressive variation was observed in all the nine families. SR had moderately high broad sense heritability (*H*^2^) across the nine families, indicating the sufficient statistical power and precision for QTL mapping and effect estimation. The highest heritability (*H*^2^ = 0.78) was estimated for family PB/KS.

YR was evaluated for 2 years in Toluca, Mexico (YR-T2010 and YR-T2011) and in Kenya (YR-K2010 and YR-K2011) (Supplementary Table 1). PBW343 had higher YR severity compared to Pavon76 (Table 3). The highest mean YR severity (%) was recorded in PB/P76. Similar to SR (Table 2), transgressive variation was observed in all the nine families. For PB/KK, *H*^2^ for YR reached the highest, 0.89, but for the other families, *H*^2^ was fairly low and could have been due to the smaller variations for disease severity between RILs in these families. LR was evaluated in Obregon, Mexico for three consecutive years (LR-2010, LR-2011, and LR-2012). PBW343 was more resistant to LR, as compared to Kenya Kudu (Table 4). Since the CIMMYT NAM population was not originally designed to study LR, fewer QTL could be identified for LR as compared with SR.

**Table 3:** **Parents' performance, means, ranges, and the heritability in the broad sense (***H***^**2**^) for yellow rust severity in nine families of the CIMMYT NAM**.

**Family**	**Parent mean**		**Progeny**					***H*^2^**
	**PBW343**	**Non-PBW343**	**No. RILs**	**No. trials**	**Mean**	**Std**.	**Range**	
PB/DZ	19.6	11.0	80	2	21.3	19.4	0–75	0.16
PB/CB	19.6	7.5	87	2	16.3	13.4	0–70	0.45
PB/JC	19.6	12.5	90	2	18.9	16.0	0–60	0.49
PB/KS	19.6	0.0	177	1	27.5	25.8	0–100	NA
PB/KB	19.6	0.5	88	2	16.7	12.4	0–50	0.29
PB/KK	19.6	0.0	89	2	20.9	25.7	0–100	0.89
PB/P76	19.6	40.0	178	1	39.9	18.4	5–100	NA
PB/MU	19.6	8.3	146	3	15.2	9.5	0–60	0.23
PB/KN	19.6	5.0	187	1	22.8	19.2	1–90	NA

*Std. is the standard deviation of the phenotype for each family*.

**Table 4:** **Parents' performance, means, ranges, and the heritability in the broad sense (***H***^**2**^) for leaf rust severity in nine families of the CIMMYT NAM**.

**Family**	**Parent mean**		**Progeny**					***H*^2^**
	**PBW343**	**Non-PBW343**	**No. RILs**	**No. trials**	**Mean**	**Std**.	**Range**	
PB/DZ	4.9	20.0	80	1	10.1	8.7	0–50	NA
PB/CB	4.9	7.5	87	2	11.8	8.4	0–40	0.10
PB/JC	4.9	15.0	90	1	8.2	8.8	0–40	NA
PB/KS	4.9	0.0	177	1	33.8	32.3	0–100	NA
PB/KB	4.9	15.0	88	1	7.8	5.3	1–30	NA
PB/KK	4.9	40.0	89	2	29.9	29.7	1–100	0.66
PB/P76	4.9	20.0	178	1	8.3	9.0	0–50	NA
PB/MU	4.9	5.0	146	1	7.4	5.4	1–30	NA
PB/KN	4.9	5.0	187	1	30.8	32.0	0–100	NA

*Std. is the standard deviation of the phenotype for each family*.

### Marker distribution on the consensus map

On the consensus map, 777 polymorphic DArT markers covered 2661.8 cM of the genetic distance of the wheat genome (Table 5; Supplementary Table 3), with an average inter-marker distance of 3.58 cM and 87.9% (683 out of 777) of unique positions (Supplementary Figures 5, 6). Due to the lack of evenly distributed polymorphic markers on wheat genome, the number of linkage groups for the consensus map was 34; there were no markers on chromosomes 1DL, 3DL, 4D, 5AL, and 5D; and less than 10 markers on each of chromosomes 1BL, 2AL, 2D, 3DL, 4AS, 4B, 5A, 6BL, and 7BS. The A, B and D genomes covered the genetic distances of 898.0 cM, 1475.0 cM, and 288.8 cM, respectively. The length of marker intervals ranged from 0 to 29.65 cM. The 489 marker intervals, corresponding to 75.4% of total marker intervals by 683 unique positions, were ranged from 0 to 5 cM in length (Supplementary Figure 6).

**Table 5:** **Summary statistics of consensus linkage map for the CIMMYT NAM population**.

**Chr**.	**Number of linkage groups**	**Number of markers**	**Number of unique positions**	**Number of markers on the short arm**	**Number of markers on the long arm**	**Genetic distance (cM)**
1A	2	59	43	22	37	193.5
1B	1	162	135	157	5	209.1
1D	1	14	14	14	0	85.3
2A	3	12	10	10	2	138.3
2B	2	50	47	29	21	272.3
2D	1	7	7	5	2	43.6
3A	3	24	23	13	11	143.7
3B	1	98	85	75	23	422.6
3D	1	26	16	26	0	6.3
4A	1	36	31	6	30	79.6
4B	2	7	7	4	3	26.6
5A	1	3	3	3	0	7.8
5B	3	42	40	18	24	311.7
6A	1	93	86	65	28	196.5
6B	2	30	30	24	6	84.2
6D	2	11	9	4	7	57.0
7A	2	30	29	18	12	138.7
7B	2	31	30	4	27	148.5
7D	3	42	38	28	14	96.7
A	13	257	225	137	120	898.0
B	13	420	374	311	109	1475.0
D	8	100	84	77	23	288.8
Total	34	777	683	525	252	2661.8

### Genetic relatedness of ten founder lines and the CIMMYT NAM population

The 10 founder lines of CIMMYT NAM population had high genetic diversity, but with different genetic distance (Figure 1). Kenya Kudu, Kenya Swara, and Kenya Nyangumi were the three varieties released in Kenya in 1966, 1972, and 1979, respectively (Table 1). Kenya Kudu and Kenya Swara shared the same origin of Ifife landrace, and Kenya Swara is in the pedigree of Kenya Nyangumi (Table 1). Therefore, the genetic distances among Kenya Kudu, Kenya Swara, and Kenya Nyangumi were closer as compared with others (Figure 1). Juchi, Kingbird, and Pavon76 were released in 1999, 1999, and 1976 from CIMMYT, Mexico. Juchi and Kingbird shared a parent Bobwhite, and Pavon76 was one of the parental lines of Kingbird (Table 1). So these three varieties are nearby in Figure 1, and Kingbird is in the middle of Juchi and Pavon76. Diniza, Crosbill, and Muu were released in 1999 from CIMMYT, Mexico. Parula is in the pedigree of Diniza, while Crosbill and Muu shared Weaver in their pedigrees, one of whose parental lines was Parula (Table 1). However, these founders were not genetically close in the plot (Figure 1), which could be partly due to the fact that the 272 SSR markers were not enough to uncover their relatedness.

**Figure 2:**
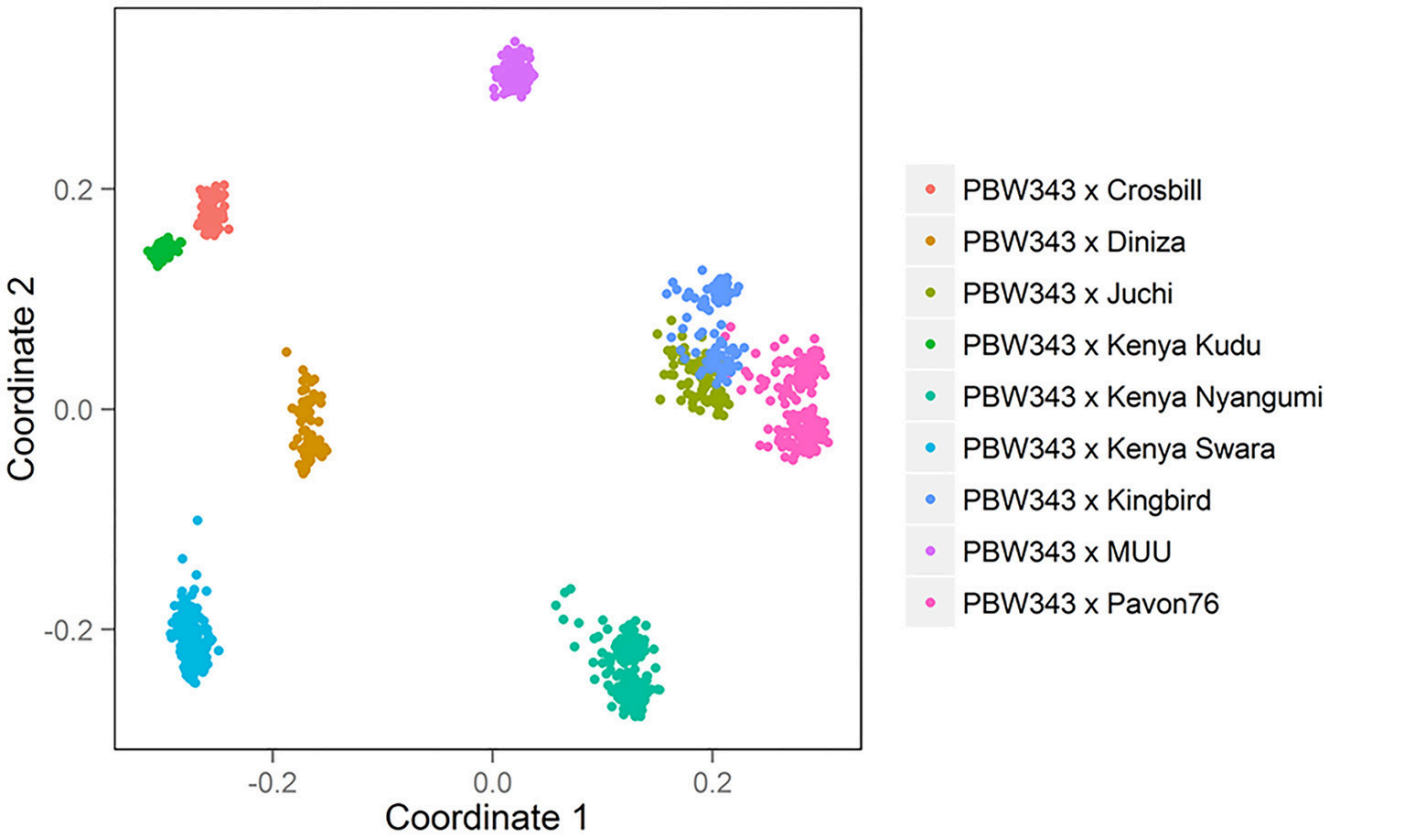
**Genetic relatedness among 1122 individuals in CIMMYT NAM population**.

The nine bi-parental RIL families can be clearly separated, except for PB/KB, PB/JC, and PB/P76 (Figure 2). From the pedigree analysis (International Wheat Information System, IWIS version 2, CIMMYT), PBW343 (ATTILA), Kingbird, Juchi, and Pavon 76 share the common origin, and three founders released from Kenya, Kenya Swara, Kenya Kudu, and Kenya Nyangumi were genetically close (Table 1). Therefore, in Figure 1 PBW343, Kingbird, Juchi, and Pavon 76 were clustered, while Kenya Swara, Kenya Kudu, and Kenya Nyangumi were grouped together. Due to the genetic relatedness of founders, the derived RIL families from PBW343, Kingbird, Juchi, and Pavon 76 had less genetic variation than these derived from PBW343, Kenya Swara, Kenya Kudu, and Kenya Nyangumi. Thus, the genetic distances among PB/KB, PB/JC, and PB/P76 were close, while the genetic distances among PB/KS, PB/KK, and PB/KN were far away (Figure 2).

### Segregation distortion loci across the whole CIMMYT NAM population

In total, 182 (23.4%) of 777 DArT markers showed evidence of segregation distortion at a 0.05 significance level. The most significant (i.e., −log*P*) segregation distortion regions (SDRs) were observed on chromosomes 7A, where −log*P* reached to 20.05, and selection favored alleles were from PBW343 (Supplementary Figure 7). Another large SDR was observed on chromosome 1B, where the selection favored alleles were from non-PBW343 parents. This region corresponds to the Rye chromosome 1RS translocation to wheat in PBW343. Some of the significant SDRs were also observed on chromosomes 1A, 6A, 7B, 3D, and 7D. No significant SDRs were found around well characterized resistance genes *Sr2*, and *Lr34*. For nine RIL families, PB/KS has the highest number of markers in segregation distortion (53.50% at a 0.05 significance level), while PB/DZ has the lowest number of segregation distortion markers (11.79% at a 0.05 significance level). For the rest of seven RILs, the averaged ratio of segregation distortion markers was 27.37% (Supplementary Tables 4–13).

### QTL controlling APR to SR, YR, and LR, and *In silico* analysis of QTL

**Figure 3:**
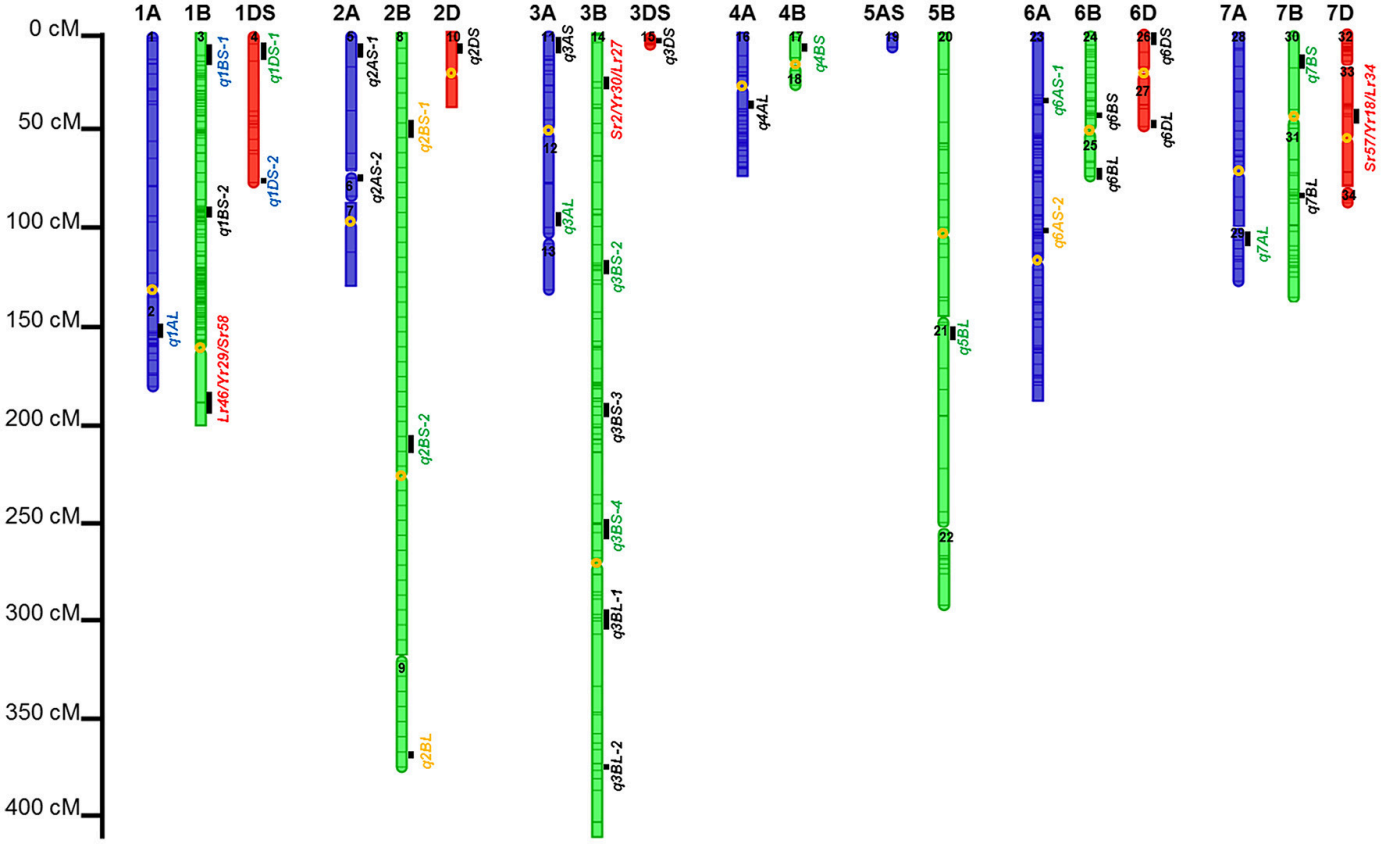
**Identified chromosomal regions harboring APR to stem, yellow, and leaf rust resistances**. Numbers in the chromosome segments were the linkage group IDs. QTL in red font were in the same regions with well characterized APR genes; in green font were the QTL regions already published but cannot be validated by *in silico* mapping; in orange font were QTL already published and also can be validated by *in silico* mapping; in blue font were novel QTL regions, and also can be validated by *in silico* mapping; and in blank font are the novel QTL regions which need to be further validated.

**Figure 4:**
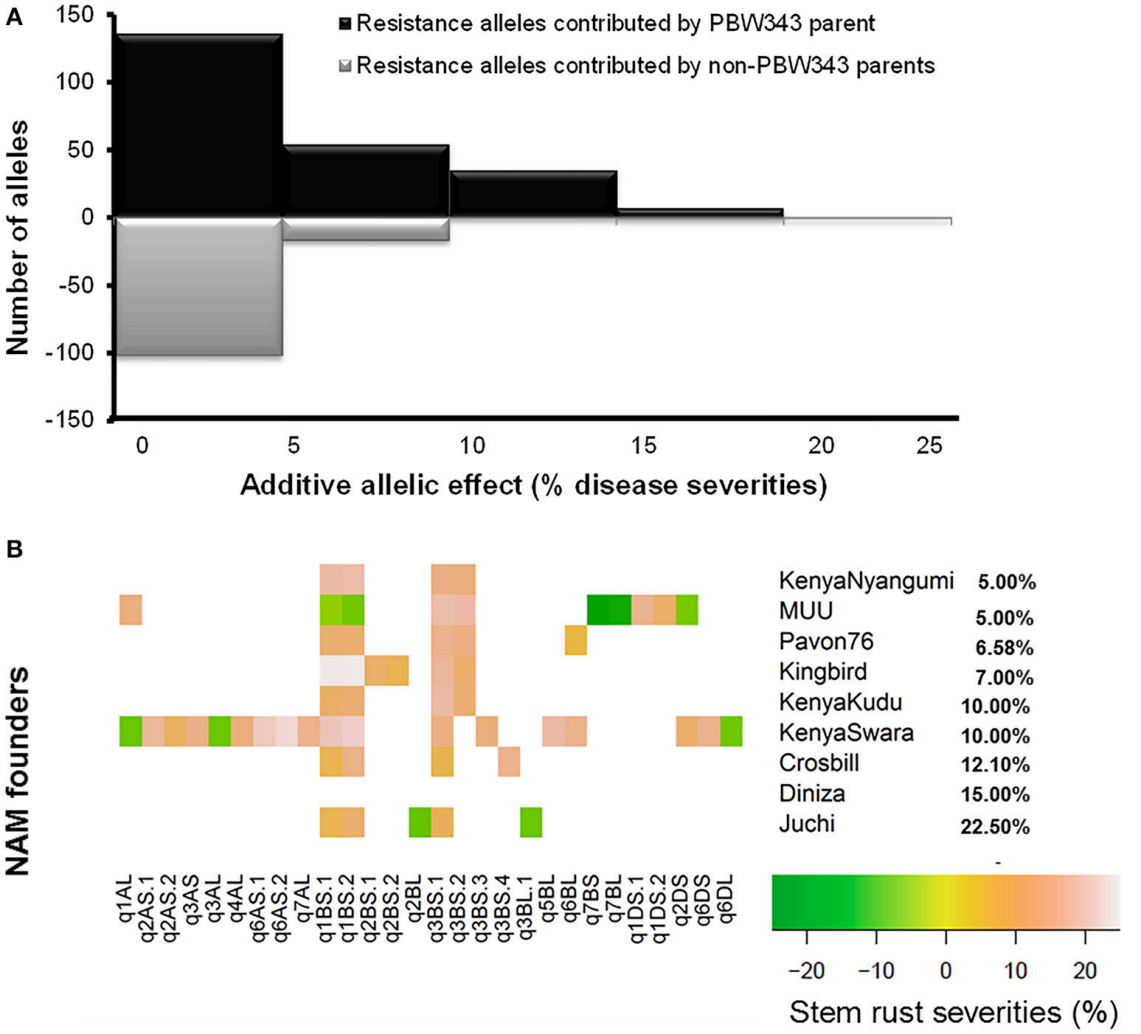
**QTL allele effect size distributions for stem rust resistance. (A)** All QTL allele effects distribution. The ratio of resistance alleles was shown above the line, and the ratio of negative alleles was shown below the line. **(B)** Heat map for significant alleles controlling stem rust resistance by QTL and allele donor. The nine APR donor lines were sorted by the phenotype of stem rust resistance.

Thirty-four identified QTL contributed to APR to SR, YR, and LR, with 9, 18, and 7 of them located on A, B, and D genomes, respectively (Tables 6–**8**; Figure 3; Supplementary Figure 8). There were 65.7, 52.2, and 57.1% of the resistance alleles were contributed by non-PBW343 parents for SR (Figure 4A), YR (Supplementary Figure 9), and LR resistance (Supplementary Figure 9), respectively. These results suggested transgressive variations for the three rust resistances in the CIMMYT NAM population (Tables 2–4; Supplementary Figure 1).

**Table 6:** **Nine QTL identified on A genome by joint inclusive composite interval mapping (JICIM) for the CIMMYT NAM population**.

**QTL**	**Trait name**	**Chr**.	**Pos. (cM)**	**Left marker^a^**	**Right marker^a^**	**Length (cM)**	**LOD**	**PVE (*%*)^b^**	**PB/DZ^c^**	**PB/CB**	**PB/JC**	**PB/KS**	**PB/KB**	**PB/KK**	**PB/P76**	**PB/MU**	**PB/KN**	**Known gene/QTL region**
*q1AL*	SR-MS2010	1AL	0	wPt-732144	wPt-732616	6.8	10.6	11.8	0.7	2.0	0.0	−5.3	−3.1	4.8	−1.0	10.7	−1.4	Validated by *in silico* mapping
	LR-2010	1AL	24	**wPt-668205**	wPt-1786	4.2	5.7	30.3	1.3	−0.8	0.5				1.2		−14.2	
	YR-T2010	1AL	24	**wPt-668205**	wPt-1786	4.2	10.9	10.8	−2.6	1.9	−1.8	−0.3	−1.7	−3.8	2.3	0.7	−10.7	
	SR-OS2010	1AL	26	wPt-664593	wPt-0432	0.6	6.3	7.7	1.8	−0.5	−1.5	−9.5	−3.2	0.8	−1.8	1.8	−5.5	
*q2AS-1*	SR-OS2010	2AS	55	**wPt-734145**	wPt-3744	26.0	5.1	13.9	3.5	−0.1	−2.7	13.3	−3.2	0.0	0.5	−1.2	0.5	
*q2AS-2*	SR-MS2010	2AS	13	wPt-743211	wPt-8068	13.4	5.6	8.9	−4.2	−3.8	1.1	8.9	5.3	−1.7	0.1	0.6	−1.8	
*q3AS*	SR-MS2010	3AS	10	wPt-1939	tPt-6949	12.3	8.9	14.1	1.1	−1.5	7.7	10.6	−6.1	−6.7	1.1	1.6	1.0	
	LR-2010	3AS	14	**wPt-0951**	tPt-0519	1.0	3.7	1.4	1.8	0.0	−3.6				0.1		0.9	
	SR-OS2010	3AS	19	wPt-1464	wPt-2748	3.7	6.2	10.7	1.0	−1.1	4.3	11.6	0.8	−1.6	−0.1	2.0	−2.4	
	YR-K2010	3AS	30	**wPt-10311**	wPt-7890	6.6	4.8	6.0	1.9	−6.0	0.0		−1.8			0.4		
*q3AL*	SR-MS2010	3AL	36	wPt-6357	wPt-9154	26.1	4.72	10.3	4.9	−1.6	−1.4	−9.3	5.2	−5.5	1.4	−0.6	0.3	CIMMYT unpublished; (Yu et al., 2014)
*q4AL*	SR-OS2010	4AL	40	wPt-744614	wPt-4424	14.3	5.17	12.2	−7.9	−1.3	1.2	10.6	−2.3	0.7	0.3	4.5	−1.1	
*q6AS-1*	SR-OS2010	6AS	57	**wPt-7623**	**wPt-3605**	0.9	12.1	21.5	−3.8	−2.0	−3.9	16.2	−1.0	3.7	−1.9	2.4	0.7	Crossa et al., 2007
	SR-MS2010	6AS	56	wPt-8539	wPt-3965	1.1	6.4	9.1	0.0	−1.7	1.1	11.3	−1.6	2.5	−0.5	2.6	2.4	
*q6AS-2*	SR-MS2010	6AS	112	wPt-6520	wPt-0832	1.0	6.3	8.2	1.9	3.0	−0.1	10.0	0.4	−0.1	−0.4	−2.3	4.3	(Yu et al., 2011); Validated by *in silico* mapping
	SR-OS2010	6AS	112	wPt-6520	wPt-0832	1.0	14.4	24.7	2.6	1.1	−3.7	17.9	−1.8	1.7	−2.5	−1.3	2.7	
*q7AL*	YR-T2010	7AL	10	wPt-3782	wPt-7763	5.9	4.0	1.9	−5.9	−0.5	0.5	−2.6	−0.6	−2.7	−2.0	0.6	0.1	CIMMYT unpublished; (Yu et al., 2014)
	SR-OS2010	7AL	31	wPt-2083	wPt-744897	8.6	5.7	13.3	1.7	−1.5	−1.6	12.0	−4.8	−2.9	−0.4	−2.2	−0.7	

a Marker name in bold means this marker was also detected by single family mapping;

b PVE, the phenotypic variance explained;

c *PB/DZ, PB/CB, PB/JC, PB/KS, PB/KB, PB/KK, PB/P76, PB/MU, and PB/KN were the RIL populations derived by PBW343 and Diniza, PBW343 and Crosbill, PBW343 and Juchi, PBW343 and Kenya Swara, PBW343 and Kingbird, PBW343 and Kenya Kudu, PBW343 and Pavon76, PBW343 and MUU, and PBW343 and Kenya Nyangumi, respectively. Underlined values were significant additive effects. Blanks means additive effects cannot be estimated, since there was no phenotypic data under the corresponding family. LOD threshold of 4.0 was used to report QTLs and determine common QTL across trials and populations. QTL having LOD score in the range of 3.0–4.0, and with pleiotropic effect with other QTL having LOD score higher than 4.0, were also reported*.

Six out of the 34 QTL had pleiotropic effects on SR, YR, and LR resistances; eight QTL had pleiotropic effects on SR and YR resistances; four QTL had pleiotropic effects on SR and LR resistances; and two QTL had pleiotropic effects on YR and LR resistances. Of the 34 QTL, three QTL were identified in regions where well characterized APR genes (marked in red in Figure 3), have been reported earlier. These QTL were *q1BL, q3BS-1*, and *q7DS* in the known genomic regions of *Sr58/Yr29/Lr46, Sr2/Yr30/Lr27*, and *Sr57/Yr18/Lr34*, respectively (Tables 6–**8**). Among them, *q3BS-1*, overlaps the gene *Sr2/Yr30/Lr27* region on chromosome 3BS (Table 7), was the largest one, explaining up to 43.7% of the phenotypic variance. This chromosome region had pleiotropic effects on SR, YR, and LR resistances, and the significant resistance alleles were contributed by non-PBW343 parents.

**Table 7:** **Eighteen QTL identified on B genome by joint inclusive composite interval mapping (JICIM) for the CIMMYT NAM population**.

**QTL**	**Trait name**	**Chr**.	**Pos. (cM)**	**Left marker^a^**	**Right marker^a^**	**Length (cM)**	**LOD**	**PVE (%)^b^**	**PB/DZ^c^**	**PB/CB**	**PB/JC**	**PB/KS**	**PB/KB**	**PB/KK**	**PB/P76**	**PB/MU**	**PB/KN**	**Known gene/QTL region**
*q1BS-1*	SR-OS2010	1BS	14	**wPt-2389**	wPt-9639	3.2	32.4	18.8	2.8	6.3	7.0	13.6	8.0	2.8	9.5	−6.1	8.7	Validated by *in silico* mapping
	YR-T2010	1BS	33	tPt-0325	wPt-8177	1.3	8.2	11.6	1.3	−0.6	−0.9	−7.6	0.7	−11.0	3.1	0.3	2.3	
	SR-MS2010	1BS	38	wPt-668076	wPt-8682	1.5	53.0	27.9	2.5	7.0	6.1	15.8	19.4	9.3	8.2	−3.7	14.1	
*q1BS-2*	YR-T2010	1BS	119	wPt-3266	wPt-0595	4.8	7.9	10.6	2.3	−1.8	0.7	−6.9	0.6	−9.8	3.1	−0.1	3.1	
	SR-MS2010	1BS	131	**wPt-5740**	wPt-7905	0.9	66.4	18.7	2.9	11.7	9.4	16.9	19.6	10.0	8.6	5.6	14.5	
	SR-OS2010	1BS	133	wPt-2075	wPt-744960	1.3	39.7	26.4	3.3	5.6	8.5	15.0	8.6	2.8	9.7	−8.8	9.7	
*q1BL*	LR-2010	1BL	181	wPt-742017	**wPt-2526**	27.6	5.1	23.4	0.2	0.7	−3.1				−2.4		−13.0	*Sr58/Yr29/Lr46*
	YR-T2010	1BL	185	wPt-742017	**wPt-2526**	27.6	9.7	31.6	2.1	−3.3	3.8	1.1	2.45	−24.9	2.9	0.8	−4.4	
	YR-T2010	1BL	200	wPt-0944	**wPt-7066**	12.3	9.0	9.6	0.8	−3.5	3.3	−5.1	1.7	−12.2	0.3	0.7	−4.0	
*q2BS-1*	LR-2010	2BS	45	**wPt-1964**	wPt-8398	16.4	6.6	4.6	−0.2	−0.2	7.4				1.4		2.9	(Yu et al., 2011); Validated by *in silico* mapping
	SR-MS2009	2BS	46	**wPt-1964**	wPt-8398	16.4	5.5	12.2		3.9			9.3	3.3	1.0	3.0		
*q2BS-2*	LR-2010	2BS	127	**wPt-1646**	**wPt-2327**	11.5	5.5	7.5	0.3	−1.2	6.3				1.1		−4.9	Njau et al., 2013
	YR-T2010	2BS	122	wPt-0489	**wPt-1646**	4.2	9.8	5.8	−5.4	−0.4	−1.8	−1.5	0.7	7.6	−0.1	−3.2	−4.0	
	SR-MS2009	2BS	110	wPt-4125	wPt-0094	9.7	5.0	10.7		3.6			7.5	1.5	−0.1	−2.6		
*q2BL*	SR-MS2010	2BL	26	wPt-7829	**wPt-2724**	2.9	8.3	5.6	3.0	0.3	−9.9	1.01	0.1	−3.9	2.1	−1.9	−1.8	(Kaur et al., 2009); Validated by *in silico* mapping
*q3BS-1*	YR-K2011	3BS	28	**wPt-666139**	**wPt-3921**	3.0	3.3	10.8								2.1		*Sr2/Yr30/Lr27* (Kaur et al., 2009); Validated by *in silico* mapping
	SR-MS2010	3BS	34	**wPt-3921**	**wPt-800213**	8.9	50.0	19.1	−5.5	1.5	9.0	10.1	13.2	12.1	12.0	14.5	11.3	
	LR-2010	3BS	35	**wPt-3921**	**wPt-800213**	8.9	4.3	3.8	0.0	0.2	−6.1				−0.8		2.0	
	YR-K2010	3BS	38	**wPt-800213**	**wPt-3609**	1.8	5.4	8.4	−1.6	4.0	0.9		6.9			0.4		
	SR-MS2009	3BS	39	**wPt-800213**	**wPt-3609**	1.8	20.5	25.9		1.6			12.6	5.5	6.7	3.5		
	YR-T2010	3BS	39	**wPt-800213**	**wPt-3609**	1.8	9.4	2.3	2.7	1.7	0.3	5.3	4.4	−0.1	0.2	1.3	0.8	
	SR-MS2011	3BS	40	**wPt-3761**	**wPt-2757**	8.2	23.1	43.7		6.9						11.5		
	SR-OS2010	3BS	41	**wPt-3761**	**wPt-2757**	8.2	53.6	15.6	−5.1	1.3	8.0	11.6	11.2	14.0	9.6	10.5	10.7	
*q3BS-2*	YR-T2010	3BS	99	wPt-743847	**wPt-1081**	6.2	5.9	1.8	−0.6	2.1	−3.2	1.4	3.4	2.3	2.2	1.2	−0.8	Njau et al., 2013
	SR-MS2009	3BS	104	**wPt-1081**	**wPt-9066**	9.7	17.3	23.4		−0.9			9.8	5.0	7.8	3.5		
	SR-OS2010	3BS	107	**wPt-1081**	**wPt-9066**	9.7	29.3	10.3	0.9	−3.3	5.4	3.8	9.1	10.0	9.8	10.2	7.3	
	SR-MS2010	3BS	108	**wPt-1081**	**wPt-9066**	9.7	33.1	16.0	0.8	−0.8	4.25	1.92	9.8	9.8	11.5	14.1	10.6	
	YR-K2011	3BS	111	**wPt-1081**	**wPt-9066**	9.7	3.7	12.7								2.3		
	SR-MS2011	3BS	114	**wPt-9066**	**wPt-740604**	9.9	16.3	45.8		−1.3						10.9		
*q3BS-3*	SR-OS2010	3BS	186	wPt-664393	wPt-9170	0.4	9.4	10.6	5.8	−0.1	4.2	10.6	−5.8	5.5	−1.0	1.3	1.7	
*q3BS-4*	LR-2010	3BS	233	rPt-5396	wPt-5786	22.5	5.5	4.0	1.7	1.2	7.0				−0.8		−0.3	Yu et al., 2011
	SR-MS2010	3BS	259	wPt-10142	wPt-7786	10.1	12.6	4.7	3.8	12.2	7.2	3.7	4.5	5.0	3.0	3.8	1.6	
	YR-T2010	3BS	261	wPt-4364	wPt-1940	0.6	15.9	14.0	1.4	−0.1	−0.4	3.6	−0.5	2.1	−12.0	−0.5	0.5	
*q3BL-1*	LR-2010	3BL	328	**wPt-11278**	**wPt-0021**	27.7	4.5	3.8	−0.4	−0.9	6.9				0.4		1.4	
	SR-OS2010	3BL	346	**wPt-0021**	wPt-9368	13.8	9.5	13.3	3.0	−6.6	−9.9	9.58	1.2	−0.5	−0.9	1.5	2.6	
*q3BL-2*	YR-K2010	3BL	384	**wPt-6834**	**wPt-6131**	3.6	5.4	11.2	3.9	−0.1	−0.6		−8.0			−0.4		
*q4BS*	LR-2010	4BS	5	wPt-5559	wPt-4607	13.2	7.6	5.0	0.8	0.3	7.5				−0.5		2.9	Kaur et al., 2009
*q5BL*	LR-2010	5BL	9	**wPt-5896**	wPt-1304	24.6	11.2	11.4	0.3	1.8	7.3				−2.6		−6.1	Kaur et al., 2009
	SR-OS2010	5BL	20	**wPt-5896**	wPt-1304	24.6	5.4	17.7	1.3	4.3	−1.8	13.9	−2.2	−1.1	1.3	−2.1	−4.2	
*q6BS*	LR-2010	6BS	55	wPt-5971	wPt-2964	5.4	6.9	2.2	1.5	−0.3	−4.9				−0.7		−1.5	
*q6BL*	SR-MS2010	6BL	6	wPt-2164	wPt-3168	4.2	6.6	6.7	2.6	−1.2	−4.2	8.4	−0.5	−0.9	2.5	2.1	4.4	
	SR-OS2010	6BL	9	wPt-2164	wPt-3168	4.2	6.1	11.6	0.9	−2.4	−2.2	12.1	−3.2	−1.8	1.5	1.3	1.0	
	YR-T2010	6BL	16	wPt-743099	**wPt-8721**	17.6	7.5	2.6	4.2	0.1	1.0	3.8	1.0	−3.4	1.7	3.9	2.5	
	SR-MS2009	6BL	21	wPt-743099	**wPt-8721**	17.6	3.8	8.0		−0.6			1.7	−1.2	5.6	1.4		
*q7BS*	SR-OS2010	7BS	24	wPt-0138	**wPt-1541**	26.2	9.5	31.1	0.0	−3.8	1.9	6.9	4.0	−0.8	−3.3	−20.2	−1.4	Yu et al., 2011; Ghazvini et al., 2012
	YR-T2010	7BS	29	wPt-0138	**wPt-1541**	26.2	5.3	3.7	−2.1	−0.6	0.8	−0.8	−2.5	−0.9	−4.9	−7.1	−0.6	
*q7BL*	SR-OS2010	7BL	44	wPt-664219	**wPt-0194**	12.5	7.1	23.2	2.6	−3.5	−0.2	−3.5	−0.9	4.7	2.5	−18.6	−0.8	
	LR-2011	7BL	45	wPt-664219	**wPt-0194**	12.5	4.2	17.1		−0.2			−0.7	3.7		−4.9		
	YR-T2010	7BL	45	wPt-664219	**wPt-0194**	12.5	6.1	9.4	6.8	−0.9	−1.9	5.5	−0.5	5.6	0.1	−6.6	−0.7	

a Marker name in bold means this marker was also detected by single family mapping;

b PVE, the phenotypic variance explained;

c *PB/DZ, PB/CB, PB/JC, PB/KS, PB/KB, PB/KK, PB/P76, PB/MU, and PB/KN were the RIL populations derived by PBW343 and Diniza, PBW343 and Crosbill, PBW343 and Juchi, PBW343 and Kenya Swara, PBW343 and Kingbird, PBW343 and Kenya Kudu, PBW343 and Pavon76, PBW343 and MUU, and PBW343 and Kenya Nyangumi, respectively. Underlined values were significant additive effects. Blanks means additive effects cannot be estimated, since there was no phenotypic data under the corresponding family. LOD threshold of 4.0 was used to report QTLs and determine common QTL across trials and populations. QTL having LOD score in the range of 3.0–4.0, and with pleiotropic effect with other QTL having LOD score higher than 4.0, were also reported*.

Thirteen QTL were in the same regions with QTL published or reviewed before (Rosewarne et al., 2013; Yu et al., 2014). Three of them (i.e., *q6AS-2, q2BS-1*, and *q2BL*, marked in orange font in Figure 3) were validated by *In silico* analysis in this study by blasting the sequences of the QTL flanking markers against to the NCBI database querying wheat (*Triticum aestivum* L.), *Brachypodium* (*Brachypodium distachyon* (L.) P. Beauv), *Hordeum vulgare* L., and rice (*Oryza sativa* L.) databases. The marker wPt-730591 can be mapped to SR resistance protein (Rpg1) gene and *Triticum turgidum subsp. durum* defense precursor (PRPI-10) gene (Supplementary Table 14); and the marker wPt-730591 was 1.79 cM up-stream of the marker wPt-6520, which was the left flanking marker of *q6AS-2* (Table 6; Supplementary Table 3). In this sense, *q6AS-2* was mapped onto rust resistance gene regions. Its significant resistance alleles were contributed by Kenya Swara (Table 6). The significant resistance alleles of most of the 10 published QTL, marked in green font in Figure 3, were contributed by non-PBW343 parents.

Eighteen QTL are not published yet, which were viewed as novel QTL detected by the CIMMYT NAM population in this study. Three of them (i.e., *q1AL, q1BS-1*, and *q1DS-2* marked in blue in Figure 3) were well confirmed by *In silico* mapping. *q1AL* had pleiotropic effects on SR, YR, and LR resistance, and explained 7.7–30.3% of the phenotypic variance. One of the salient features of NAM design is that we could order the resistance alleles by common parent's allele as reference (Buckler et al., 2009). For *q1AL*, its resistance alleles from strong to weak can be ordered as Muu allele, PBW343 allele and Kenya Swara allele. That is to say, compared with Kenya Swara and Muu at this locus, the resistance alleles came from Muu with size 20.2 (i.e., 10.7 + 9.5 in Table 6), which is consistent with the SR resistance phenotype of Kenya Swara and Muu (Table 2). The resistance alleles controlling YR and LR resistance of *q1AL* were all contributed by PBW343 in PB/KN. Fifteen out of 18 novel QTL need to be validated further (marked in black font in Figure 3). Seven of them were pleiotropic QTL (i.e., *q3AS, q3BS-4, q3BL-1, q6BL, q3DS*, and *q6DL*).

In general, single family linkage analysis has less precision and statistical power than joint linkage analysis for identifying common QTL (Li et al., 2011). In the present study, 21 QTL (61.7%) identified by joint linkage mapping (Tables 6–8) were also identified by single family mapping. The number of significant QTL identified in each family was 7, 8, 14, 23, 7, 30, 9, 17, and 13 for PB/DZ, PB/CB, PB/JC, PB/KS, PB/KB, PB/KK, PB/P76, PB/MU, and PB/KN, respectively (Supplementary Table 15). The highest number of QTL were detected in PB/KK, maybe due to the large genetic distance between PBW343 and Kenya Kudu (Figure 1), and the large phenotypic distance between PBW343 and Kenya Kudu and the large phenotypic variance in their RIL progenies for all three rust resistance traits (Tables 2–4).

**Table 8:** **Seven QTL identified on D genome by joint inclusive composite interval mapping (JICIM) for the CIMMYT NAM populations**.

**QTL**	**Trait Name**	**Chr**.	**Pos. (cM)**	**Left Marker^a^**	**Right Marker^a^**	**Length (cM)**	**LOD**	**PVE (%)^b^**	**PB/DZ^c^**	**PB/CB**	**PB/JC**	**PB/KS**	**PB/KB**	**PB/KK**	**PB/P76**	**PB/MU**	**PB/KN**	**Known gene/QTL region**
*q1DS-1*	YR-T2010	1DS	11	**rPt-4471**	**wPt-5320**	16.8	6.4	14.0	1.6	1.7	−2.0	−10.3	−0.1	4.4	2.6	0.1	5.1	Njau et al., 2013
	SR-MS2010	1DS	32	**wPt-7140**	wPt-671545	7.0	13.7	9.1	0.8	1.5	3.0	5.5	−0.8	2.3	−0.7	12.5	3.3	
*q1DS-2*	SR-MS2010	1DS	74	wPt-1387	wPt-7953	10.1	5.7	4.4	1.4	1.1	2.0	0.2	1.4	2.8	0.1	9.2	2.2	Validated by *in silico* mapping
*q2DS*	SR-MS2010	2DS	21	wPt-2644	wPt-667584	18.9	9.6	13.9	1.5	−2.3	5.4	9.7	3.9	2.1	−0.5	−8.2	0.4	
*q3DS*	LR-2010	3DS	5	wPt-740602	wPt-742368	0.5	5.5	20.4	1.6	0.1	−0.9				−0.8		11.8	
	YR-T2010	3DS	5	wPt-740602	wPt-742368	0.5	3.3	5.6	−0.2	−0.2	1.7	−7.8	−0.4	−0.7	−0.5	1.5	0.9	
*q6DS*	SR-MS2010	6DS	0	wPt-667005	wPt-3879	11.0	5.9	7.6	1.8	−3.3	−0.4	9.2	4.2	−1.0	2.2	−2.4	1.9	
	SR-OS2010	6DS	23	wPt-3879	wPt-741955	12.4	7.9	13.4	5.2	−0.3	−0.7	12.1	5.4	1.4	−0.2	−5.1	2.3	
*q6DL*	SR-MS2010	6DL	0	**wPt-3127**	wPt-731465	5.6	4.8	7.2	−1.4	−3.9	0.8	−8.9	1.5	−0.7	1.7	1.6	−3.1	
	LR-2012	6DL	19	**wPt-731605**	**wPt-668152**	2.7	10.3	30.8				16.3		−8.3				
	LR-2011	6DL	26	**wPt-668152**	**wPt-665675**	12.2	5.3	42.7		0.5			1.2	−11.4		1.1		
*q7DS*	SR-OS2010	7DS	29	wPt-7508	**wPt-4555**	28.5	3.7	4.0	0.7	−1.7	0.6	4.8	5.4	0.8	2.6	−1.8	−2.4	*Sr57/Yr18/Lr34*
	YR-T2010	7DS	42	wPt-7508	**wPt-4555**	28.5	5.4	9.7	−1.9	1.4	−2.1	6.8	1.9	−6.8	5.8	−1.8	3.3	

a Marker name in bold means this marker was also detected by single family mapping;

b PVE, the phenotypic variance explained;

c *PB/DZ, PB/CB, PB/JC, PB/KS, PB/KB, PB/KK, PB/P76, PB/MU, and PB/KN were the RIL populations derived by PBW343 and Diniza, PBW343 and Crosbill, PBW343 and Juchi, PBW343 and Kenya Swara, PBW343 and Kingbird, PBW343 and Kenya Kudu, PBW343 and Pavon76, PBW343 and MUU, and PBW343 and Kenya Nyangumi, respectively. Underlined values were significant additive effects. Blanks means additive effects cannot be estimated, since there was no phenotypic data under the corresponding family. LOD threshold of 4.0 was used to report QTLs and determine common QTL across trials and populations. QTL having LOD score in the range of 3.0–4.0, and with pleiotropic effect with other QTL having LOD score higher than 4.0, were also reported*.

Regarding effect estimation, joint linkage analysis allowed us to estimate a separate effect at each QTL for all nine families (Tables 6–8; Figure 4). The SR resistance varied by 60% among 10 APR donors, and by 100% among the whole NAM population (Table 2); the YR resistance varied by 40% among 10 donors, and by 100% among the population (Table 3); and the LR resistance varied by 40% among 10 donors, and by 100% among the population (Table 4). All the 10 parents were found to be susceptible to YR and LR at seedling stage showing score of 8 or 9 on a 0–9 scale except Crossbill for YR which scored 6 (intermediate) on the 0–9 scale. Relative to PBW343, the largest SR resistance effect of QTL allele had an additive effect of 20.2% (Figure 4A), while the largest YR and LR resistance effects were 24.9% and 16.3%, respectively (Supplementary Figure 9). A total of 56 alleles out of 362 SR resistance alleles were significant (LOD score > 2.5). The resistance significant alleles for four QTL in chromosome 3BS were all contributed by non-PBW343 parents (Figure 4B). We searched for the presence of epistatic interaction in the CIMMYT NAM population by testing all pairwise marker combinations. No significant epistasis was identified.

### Prediction

**Figure 5:**
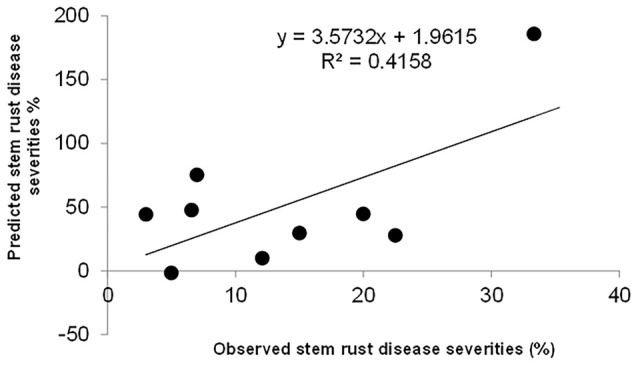
**Predicted stem rust resistances of 10 founder lines based on additive QTL model**.

The significant additive effect estimates of SR resistance QTL were able to predict parental SR by *R*^2^ = 0.41 (Figure 5). Considering that the heritability of SR across nine RIL families were in the range of 0.45–0.78 (Table 2), the prediction power was enough to provide further evidence that epistasis is relatively unimportant in this population for SR resistance. The predicted YR and LR resistances of founders from the CIMMYT NAM QTL were low (results not shown), partly because the marker density was low, and the YR and LR resistances diversities of founders for this CIMMYT NAM population was narrow (Tables 3, 4), and then did not have vigor to detect all possible QTL related to the YR and LR resistances and to estimate their effects accurately.

## Discussion

### Extensive genetic understandings of the APR donor lines

Characterizing diverse APR sources are critical to maximize the genetic variability, to produce the superior recombinant genotypes, and to pyramid the resistances into improved wheat lines. Since last century, Global Wheat Program at CIMMYT has taken efforts for breeding minor, slow-rusting genes based APR, which was the field based selection in conjunction with other traits and the high returns from investments due to long-term effectiveness. During these efforts, the nine historical APR donor lines utilized in this study, were identified to cover a wide range of APR genetic diversity, and used as one of the parents to develop genetic mapping populations in wheat. However, the genetic knowledge of the APR donors was limited to further strategize the rust management in breeding programs. The genetic relationship revealed in this study (Figure 1) showed the highly genetic similarities of three founders lines released in Kenya (i.e., Kenya Swara, Kenya Kudu, and Kenya Nyangumi). Three varieties released in Mexico, 1999 (i.e., Diniza, Juchi, and Kingbird) were more genetically similar with Pavon76, which was released in Mexico, 1976, rather than Muu, which was released at the same year and same place with Diniza, Juchi, and Kingbird (Table 1). Crosbill was genetically far away from the other eight APR donor lines and PBW343. The genetic knowledge of the APR donor lines learnt from this study would aid the identification of genotypes with promising and desirable rust resistances, and agronomic traits for hybridization in wheat breeding.

The pedigree information of the nine donors was clear and available from germplasm curator, but it does not necessarily reflect the underlying genetics (Soleimani et al., 2007). In addition, genetic relatedness calculated by pedigree information does not take into account the effects of selection, mutation and genetic drift, and requires several simplifying assumptions that are generally not met. In contrast, molecular markers allow the assessment of relatedness directly at the DNA level by estimation of the proportion of alleles that are identical by state. In this sense, the extent of the information that they can provide might depend on the nature and number of markers (e.g., level of homoplasy, mutation rate), the genome coverage and distribution, and the population under investigation (Maccaferri et al., 2003). In this study, 272 SSR markers were utilized to investigate the genetic relatedness of nine APR donors, which will be further evaluated by markers explored through genotyping-by-sequencing (Li et al., 2015).

### Utilizing the NAM genetic design to facilitate the gene identification for rust resistance

NAM design had power to reveal QTL which otherwise was undetected in previous studies (Buckler et al., 2009; Bajgain et al., 2016). Maize NAM population has been used extensively for dissection of complex traits (Buckler et al., 2009; Brown et al., 2011; Poland et al., 2011; Tian et al., 2011; Cook et al., 2012). The CIMMYT NAM population reported in this study is the largest publicly available platform for rust resistance dissection in wheat. Most recently, Bajgain et al. (2016) use a spring wheat NAM panel composed of 10 RIL families with 852 lines to conduct joint linkage analysis for SR resistance. Fifty-nine additive QTL, explaining 1–20% of the phenotypic variance were identified, and no epistatic QTL was detected. *q2AS-1, q2BL, q3AL, q4BS*, and *q5BL* identified in this study were likely in the same regions of five QTL reported by Bajgain et al. (2016). However, as indicated by Bajgain et al. (2016), due to the de novo marker system and the lack of sequence alignment for the markers they used, it is hard for us to have a position-based definitive comparisons for QTL detected by Bajgain et al. (2016) and by this study. Further, comparisons have been made with previous studies based on linked markers as presented in Tables 6–8 (reference reports presented in the last column). To facilitate this head-to-head comparison and uncover candidate genes, it is necessary to have the functional annotation and high density genomic maps for the published wheat genome. Then, more work could be done for having both traditional marker types (like SSR and DArT) and sequenced-based markers anchored to the physical map.

In this study, the successful demonstration of the power of the CIMMYT NAM population is exemplified not only by correspondence of QTL previously identified in wheat, but also by identification of novel QTL. Chromosomal regions associated with three well characterized APR genes (i.e., *Sr58/Yr29/Lr46, Sr2/Yr30/Lr27*, and *Sr57/Yr18/Lr34*) and 13 previously reported QTL were successfully identified (Tables 6–8), and 18 QTL were first detected in this study. Through *in silico* mapping, we have found that the three novel QTL showed sequence similarities with R like genes in *Triticum aestivum, Triticum turgidum* subsp. *durum, Triticum turgidum* ssp. *dicoccoides, Brachypodium distachyon, Hordeum vulgare*, and *Oryza sativa* encoding proteins (Tables 6–8). Of all the 34 QTL identified, 14 were identified by high resolution with their marker-interval lengths within 5 cM; and 20 have pleiotropic effects on SR, YR, and LR resistances. Rather than inferring multiple alleles at each testing locus as in multiple-parent design, NAM reduced the testing to exact bi-allelic contrasts across the whole population. All allele effects were estimated by PBW343 allele as a reference. Therefore, phenotypes of the CIMMYT NAM founders could be predicted by the estimated QTL allele effects adding to the observed PBW343 phenotype. The prediction ability for SR resistance QTL was 41.6%, which was close to the heritability in the broad sense of SR resistance (Table 2). This indicated that the additive QTL for SR (Tables 6–8) were reliable and epistatic variance was not significant.

### Marker density and distribution of the consensus map

The consensus map in this study was constructed by 777 DArT markers, which were polymorphic in at least three RIL families. Compared with A and D genomes, B genome revealed the maximum percentage of total and unique number of markers (54.1 and 55.8%, respectively; Table 5), the longest genetic length (1475.0 cM; Table 5), and the maximum number of detected QTL regions (18 out of 34 QTL; Table 7; Figure 3). These results were consistent with previous results (Li et al., 2015) and in accordance with previously reported genetic maps (Sansaloni et al., 2011; Cavanagh et al., 2013; Rosewarne et al., 2013; Li et al., 2014; Wang et al., 2014; Yu et al., 2014). The D genome contained 12.8% of total markers and 7 out of 34 QTL detected, which reinforced that genomic variation in the D genome of bread wheat is consistently low (Singh et al., 2013; Eckard et al., 2014; Wang et al., 2014). The number of linkage groups for the consensus map and each of the nine RIL family was 34, 20, 18, 28, 23, 23, 21, 25, 41, and 30, respectively (Table 5; Supplementary Table 3). These results were not surprising considering the lack of markers in some chromosome regions to cover the wheat genome. This also resulted in a lower phenotypic prediction accuracy of founder lines, particularly for YR and LR resistances.

On the consensus map, there are 162 markers located on chromosome 1B; 157 markers on its short arm (1BS) and 5 markers on its long arm (1BL). It is further noteworthy that all the markers on chromosome 1BS in this study were distributed on the satellite region of chromosome 1BS in wheat, and the polymorphism rates in the satellite region have been reported to be much higher than the average rate for the whole wheat genome (Zhang et al., 2000; Wilkinson et al., 2012). Also, the satellite region on the chromosome 1BS in wheat is known to contain many agronomical important genes (Zhang et al., 2000; Wilkinson et al., 2012). In this study, we found two novel pleiotropic QTL controlling SR and YR resistances on chromosome 1BS, one of which was confirmed to be located in the rust resistance gene region by *in silico* mapping (Supplementary Table 14).

### Controlled types I and II errors

Many statistical methods (Zeng, 1994; Xu, 2003; Li et al., 2007) have been proposed to control the Type I (false-positive) and Type II (false-negative) error rates while mapping multiple QTL. The simple algorithm implemented in ICIM (Li et al., 2007) and its extension JICIM (Li et al., 2011) has become the method of choice because of its fast speed, high QTL detection power (i.e., low Type II error), and low false discovery rate (i.e., low Type I error), etc. In ICIM, the largest probability for markers moving into the model (PIN) is the only subjectivity comes into play, and may have a big effect on the QTL mapping results. Here, two ways were utilized to determine the PIN in ICIM and JICIM. One is the extensive permutation tests (Anderson and ter Braak, 2003) to determine PIN (Buckler et al., 2009; Li et al., 2011) and LOD threshold to declare the existence of QTL. The other is *QQ*-plot, which has been used extensively in (genome-wide) association mapping (Yu et al., 2008; Tian et al., 2011), but has virtually no application in linkage analysis. In this study, we monitored the over-fitting of genetic models and determined the PIN under the help of *QQ*-plot (Supplementary Figures 3, 4). This offers another vision to better utilize the statistical methods for empirical data in linkage analysis.

## Conclusion

PBW343 was a popular, high-yielding modern variety, developed in the 1990s and once grown on millions of hectares in India. However, its resistance has been overcome to various rusts, including Ug99 race group of SR. Diverse sources of APR lines have been identified at CIMMYT and worked toward developing wheat varieties resistant to Ug99 by pyramiding several APR genes using molecular markers (Singh et al., 2015). In this study, we employed the analytic design NAM to unknotted APR in historical diverse parental lines with large scale of phenotyping. Thirty-four genetic loci associated with APR, 20 of them having pleiotropic effects on wheat rusts. We also identified 18 new candidate gene-regions controlling APR with large effects as compared with others. Not only the novel knowledge was gained for APR, but also the new analytical methodology for facilitating the applications of NAM design in crop genetics was suggested. Novel pleiotropic QTL found in this study enrich the genetic resources for addressing potential threat to wheat production and food security. The set of APR regions identified in this study predicted the SR resistance in wheat, will acquire a better genomic understanding of rust resistances, and will envision the future rust management strategy.

## Author contributions

Conceived and designed the experiments: SS and RS. Performed the experiments SS, SB, BB, and JH, Analyzed the data: HL, DS, JB, PV, and SS. Wrote the paper: HL, SS, DS, BB, SB, PV, and RS. All authors read and approved the final version of manuscript.

## Funding

Mapping populations' development and phenotyping for wheat rusts were supported by a grant from the Bill & Melinda Gates Foundation and DFID (UK) to Cornell University for the Durable Rust Resistance Wheat (DRRW) Project funded by BMGF [Grant ID# 49767 (PI) and 60169 (PII)]. The genotyping, analysis and preparing the research article were supported by the Seeds of Discovery project funded by the Sustainable Modernization of Traditional Agriculture (MasAgro) project supported by the Government of Mexico. Also, thanks to the Natural Science Foundation of China (No. 31471174) to support one of co-authors. We thank the editor and the reviewers for their valuable suggestions.

### Conflict of interest statement

The authors declare that the research was conducted in the absence of any commercial or financial relationships that could be construed as a potential conflict of interest.
