# Supplementary material for: Identification of Genomic Associations for Adult Plant Resistance in the Background of Popular South Asian Wheat Cultivar, PBW343
Source: Front Plant Sci. 2016 Nov 8;7:1674. doi: 10.3389/fpls.2016.01674 (PMC5099247; doi:10.3389/fpls.2016.01674)
Supplement: Supplementary file 16 [file Image1.pdf]

Supplementary Material

Uniting the historical donor lines to unravel genetics of adult plant resistance to wheat rusts by nested association mapping

Huihui Li, Sukhwinder-Singh*, Sridhar Bhavani, Ravi Prakash Singh, Deepmala Sehgal, Bhoja Raj Basnet, Prashant Vikram, Juan Burgueno-Ferreira, Julio Huerta-Espino

*** Correspondence:** Corresponding Author: suk.singh@cgiar.org

1 Supplementary Figures and Tables

1.1 Supplementary Figures

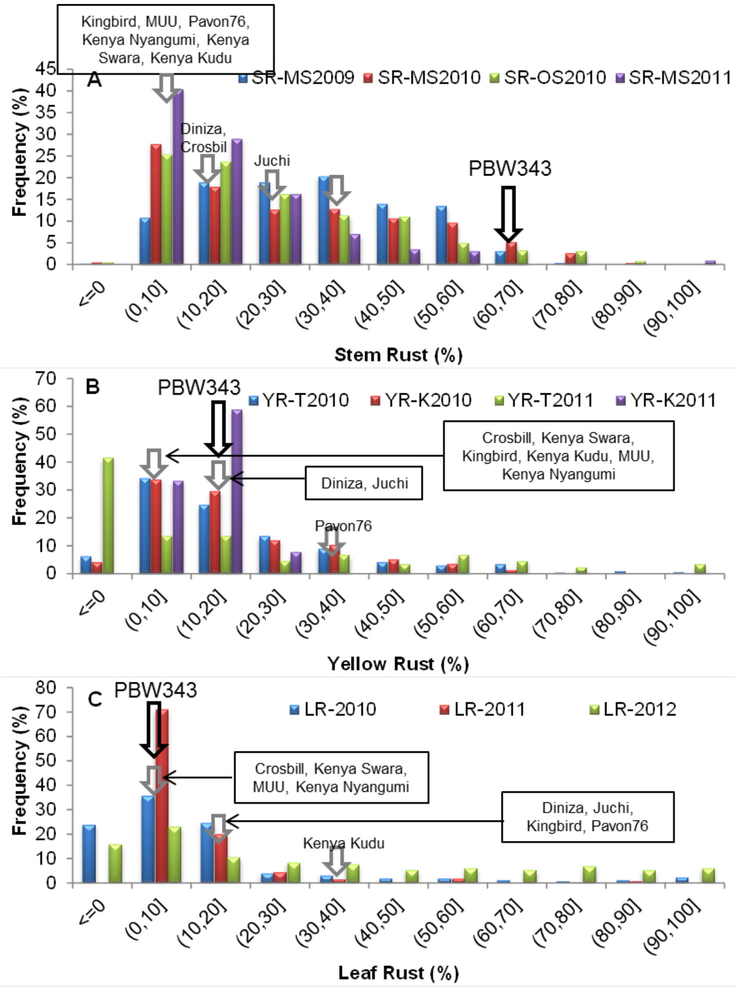

Supplementary Figure 1. Phenotypic distribution of stem rust (A), yellow rust (B), and leaf rust (C) in wheat NAM population

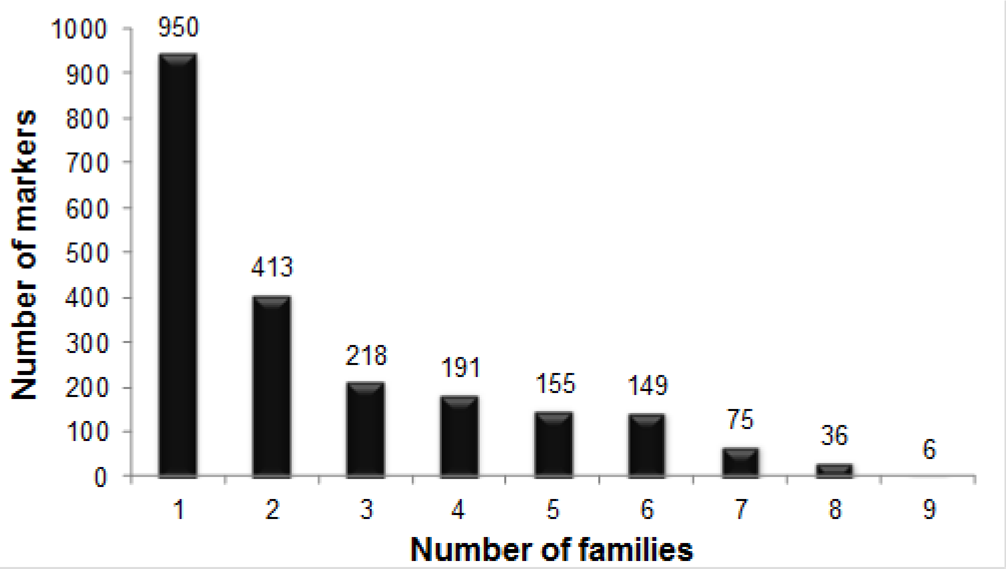

Supplementary Figure 2. The number of common markers across the nine RIL families in wheat NAM population. 950 out of 2193 markers are polymorphic in only one family, 413 markers are polymorphic in two families, and 218 markers are polymorphic in three families and so on.

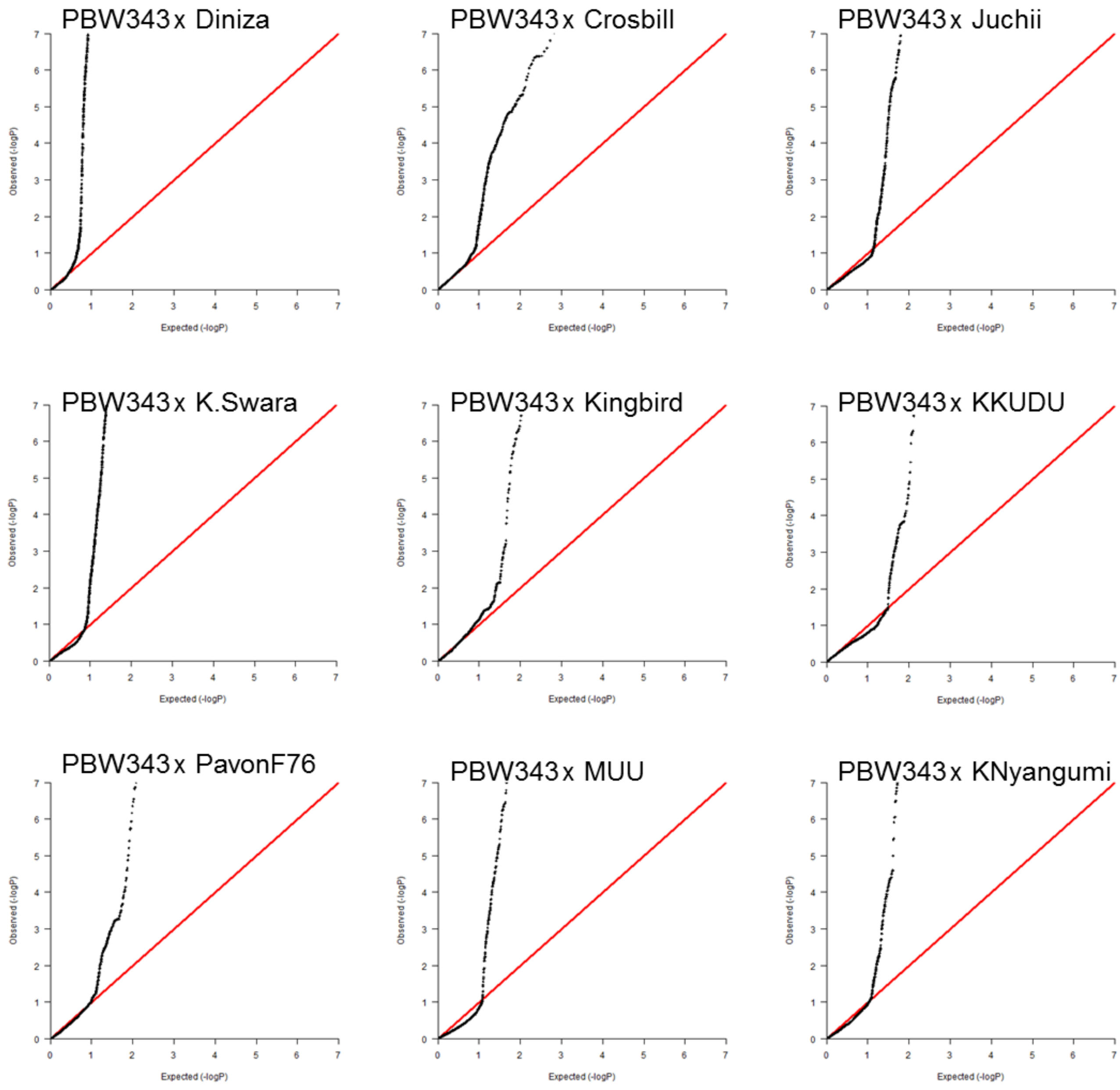

Supplementary Figure 3. *Quantile-Quantile* (QQ) plot when the probability for markers moving into the model was set as 0.05 in inclusive composite interval mapping (ICIM).

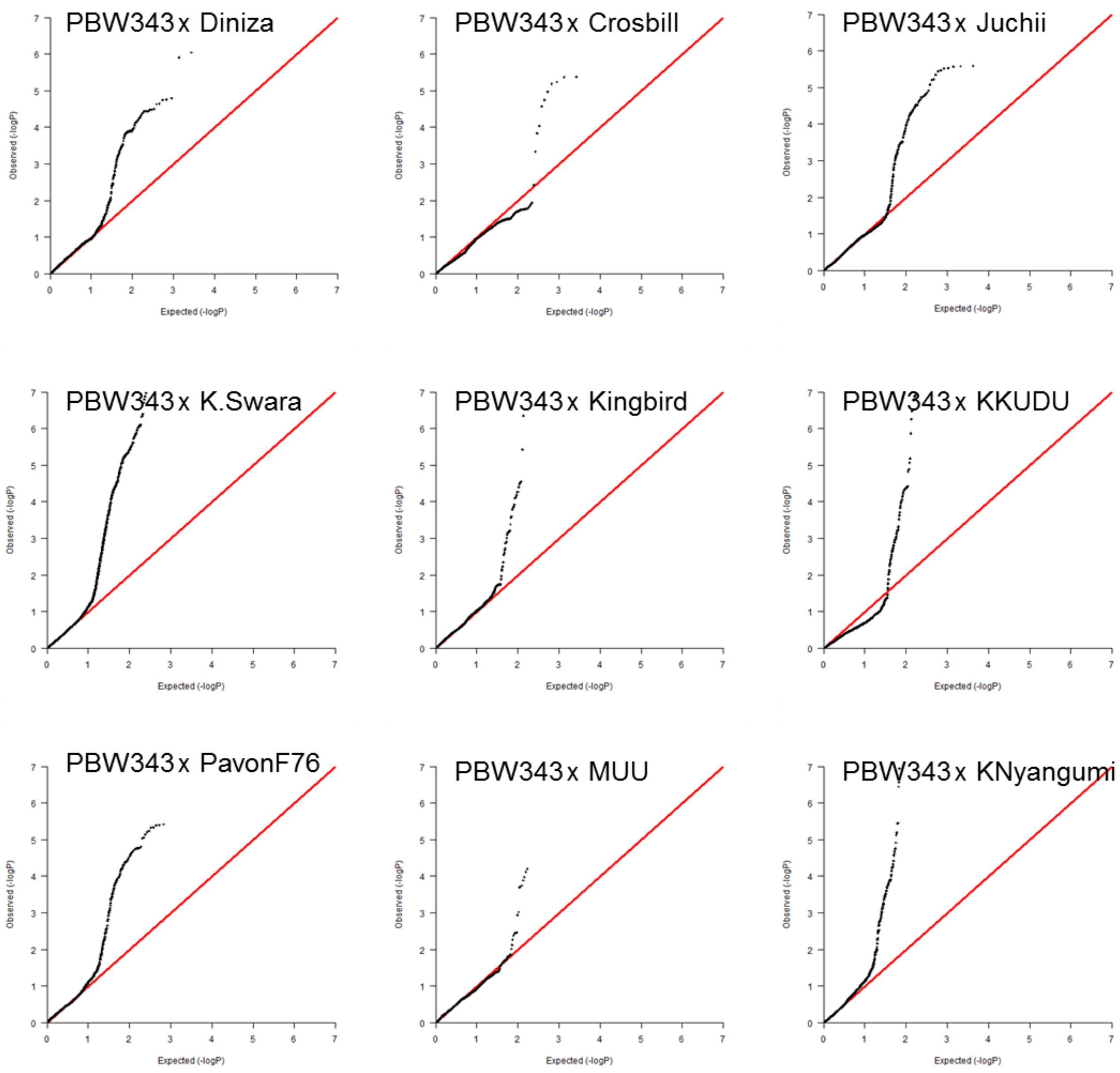

Supplementary Figure 4. *Quantile-Quantile* (QQ) plot when the probability for markers moving into the model was set as 0.01 in inclusive composite interval mapping (ICIM).

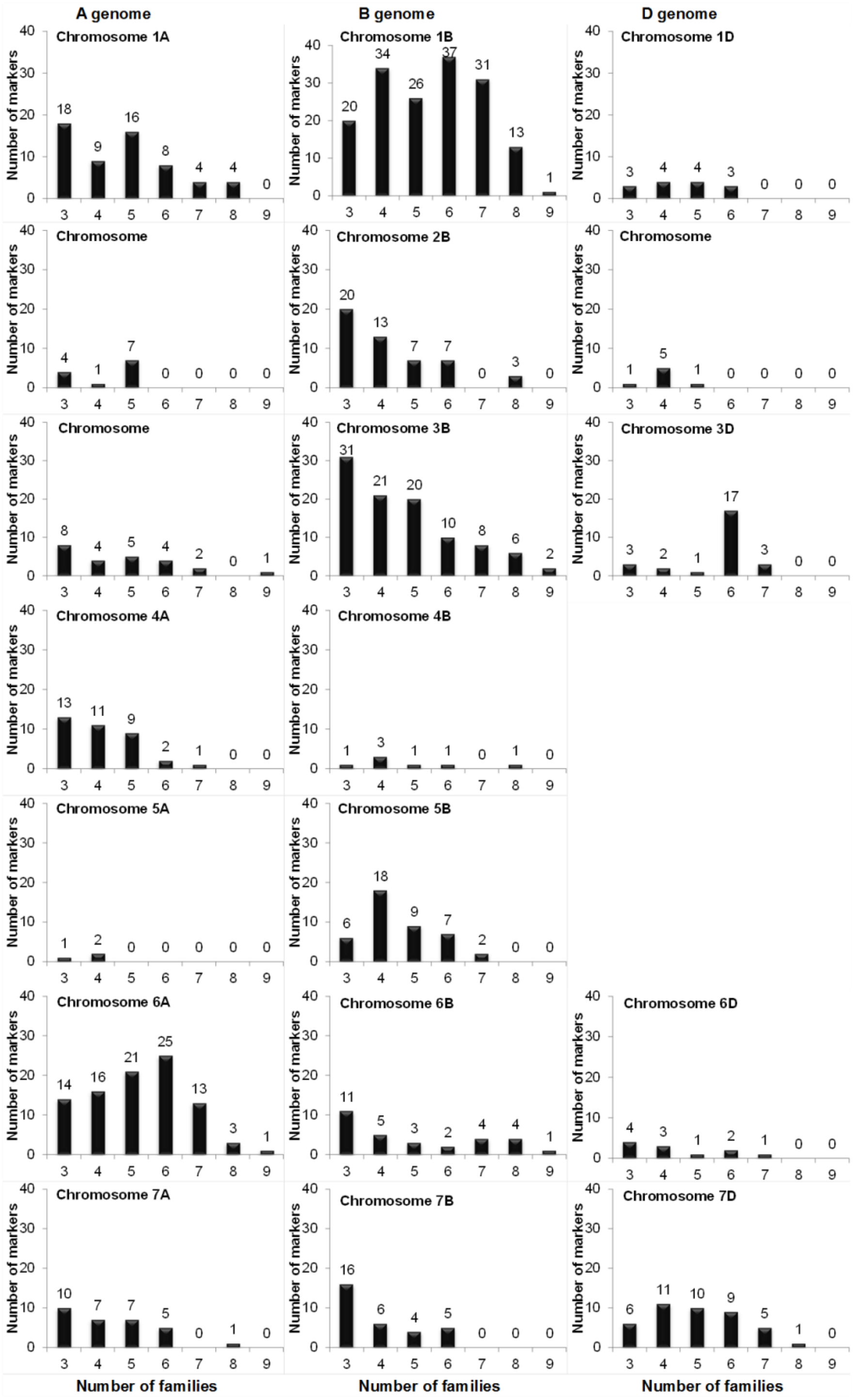

Supplementary Figure 5. The number of common markers across chromosomes for markers polymorphic in over three families.

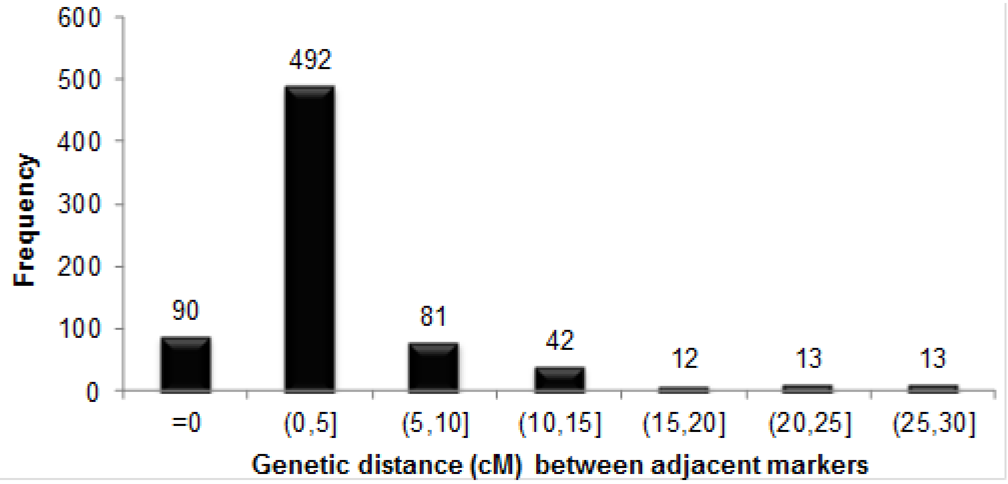

Supplementary Figure 6. The genetic distance (cM) between adjacent markers for the consensus linkage map constructed for wheat NAM population.

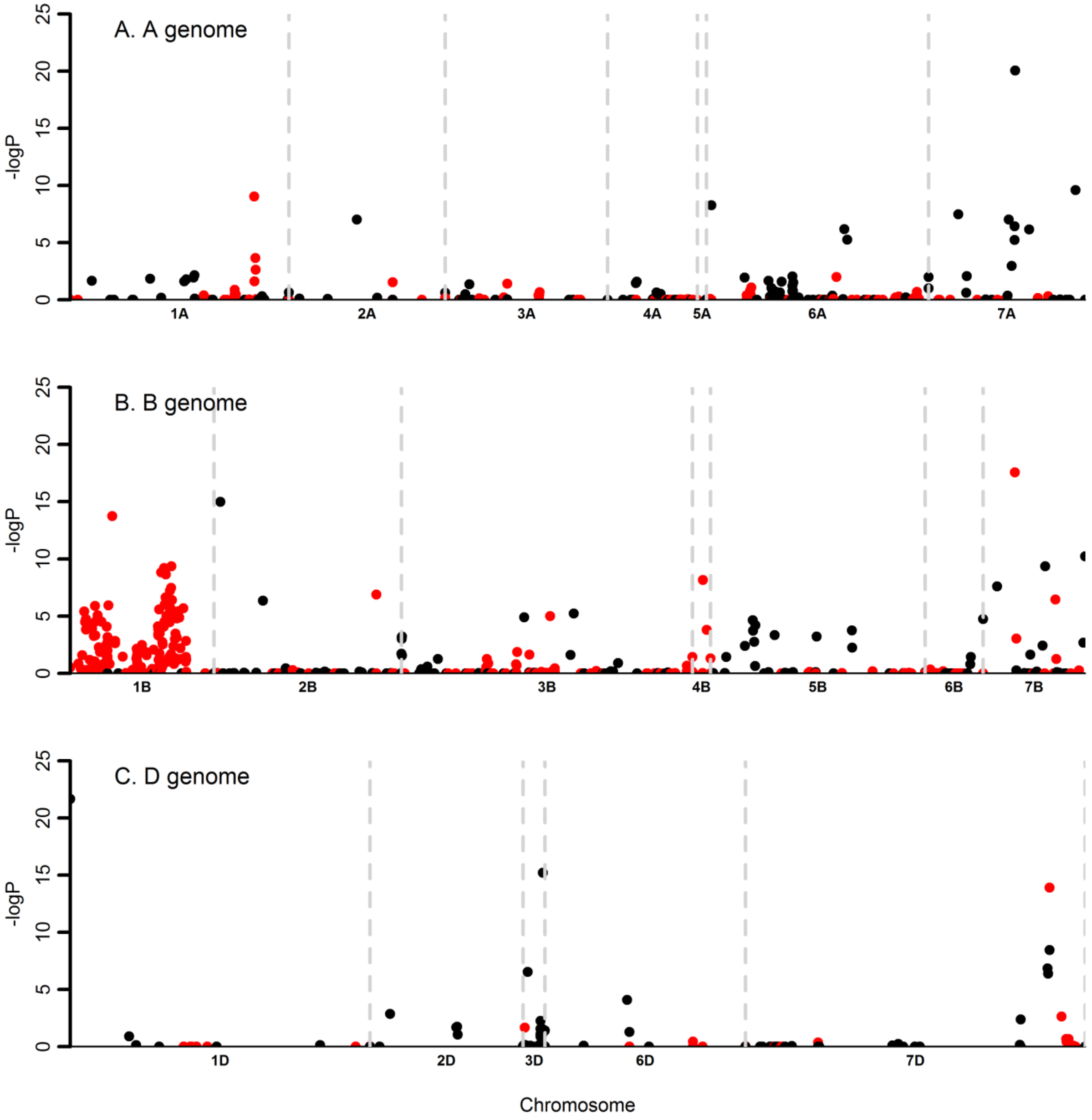

Supplementary Figure 7. Manhattan plot of segregation distortion loci mapping by consensus linkage map in wheat NAM population, A for A genome, B for B genome, and C for D genome. Red dot indicated that selection of segregation distortion favored alleles from non-PBW343 parents; while black dot indicated that selection of segregation distortion favored alleles from PBW343.

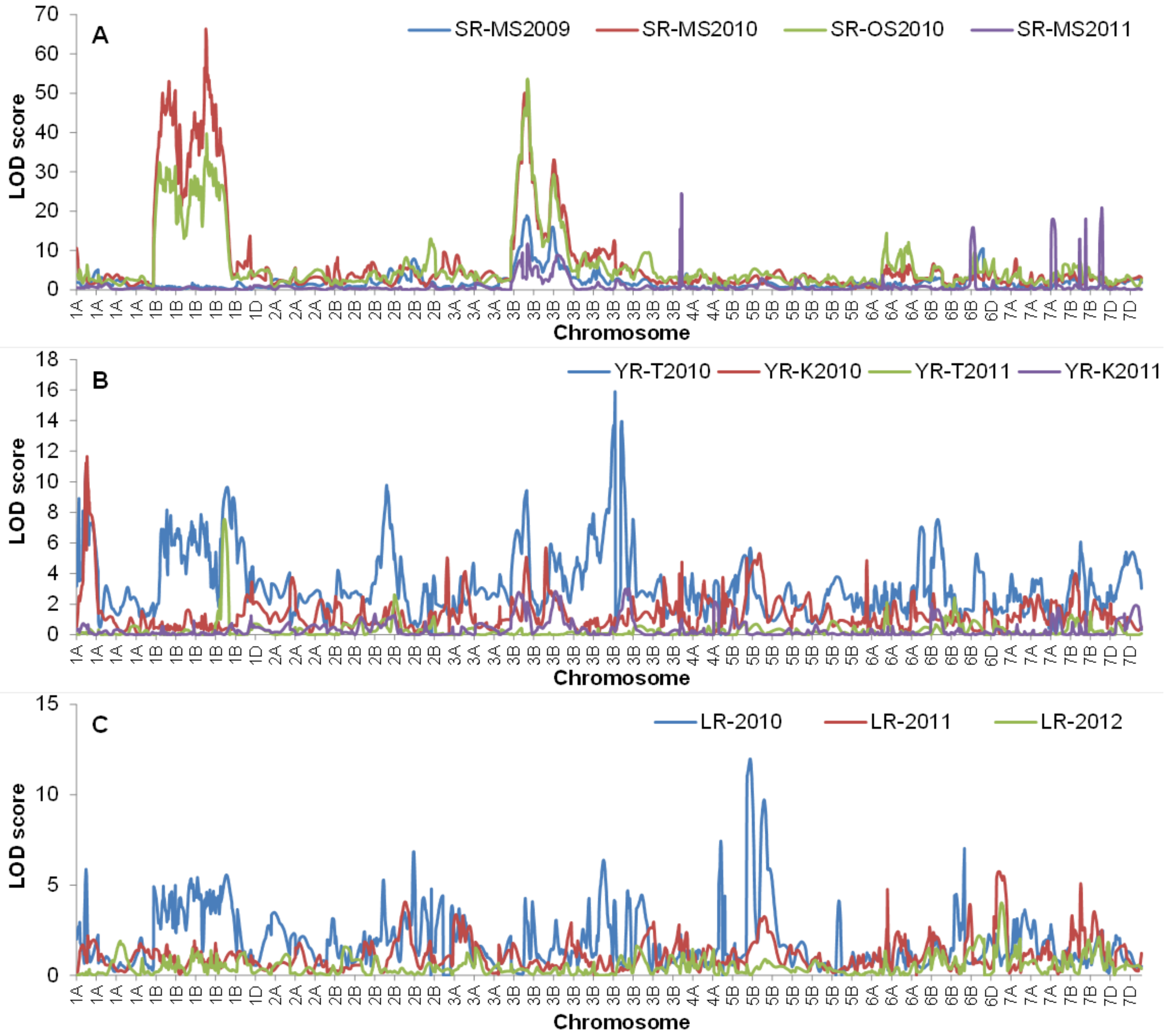

Supplementary Figure 8. LOD profile from Joint Inclusive Composite Interval Mapping (JICIM) for stem rust (A), yellow rust (B), and leaf rust (C) across trials and across wheat genome.

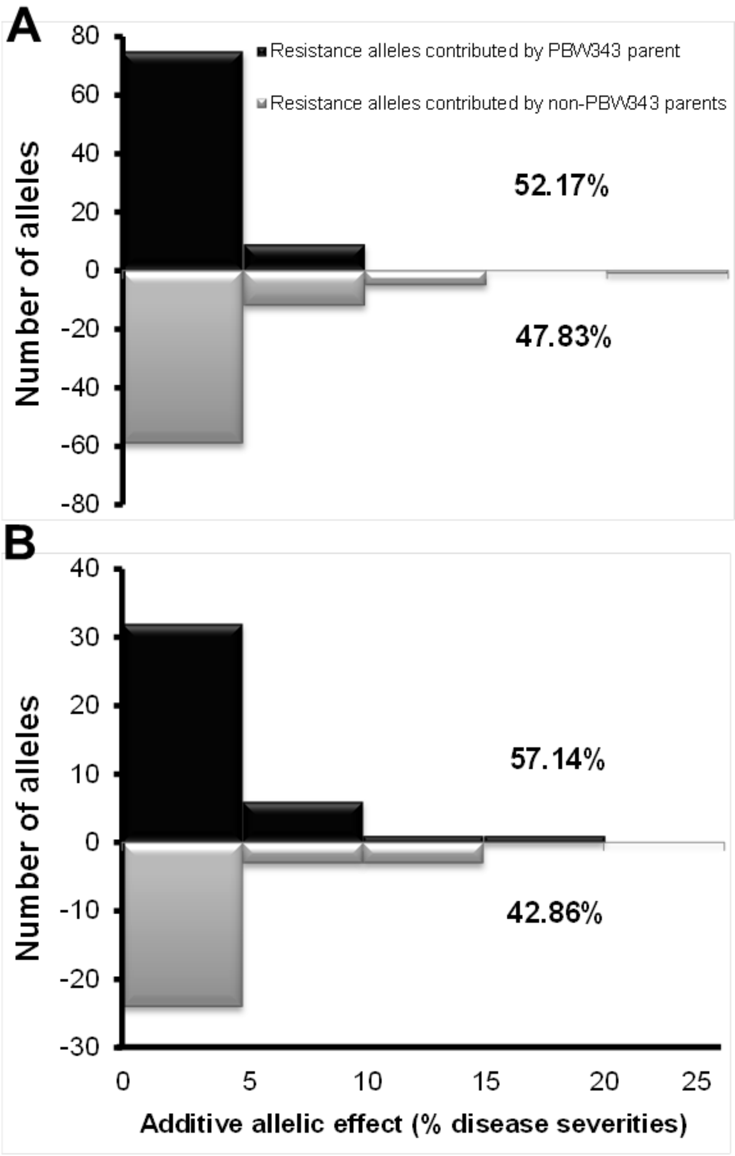

Supplementary Figure 9. All QTL allele effects distribution. The ratio of resistance alleles was shown above the line, and the ratio of negative alleles was shown below the line. A for yellow rust resistance, and B for leaf rust resistance.

1.2 Supplementary Tables

Supplementary Table 1. Traits measurements across nine RIL families
Supplementary Table 2. 272 SSR markers used to calculate the similarity among ten founders
Supplementary Table 3. Consensus linkage map of wheat NAM population
Supplementary Table 4. Information on nine linkage maps across nine RIL families

Supplementary Table 5-13. Linkage maps of nine families
Supplementary Table 14. Results from *in silico* mapping
Supplementary Table 15. QTL identified from single family QTL mapping by inclusive composite interval mapping (ICIM)
